# Florigen and Florigen‐Like Genes Regulate Temperature‐Responsive Flowering in Tomato

**DOI:** 10.1002/advs.202506711

**Published:** 2025-07-24

**Authors:** Jia Song, Shiqi Zhao, Siyu Fang, Xiaotian Wang, Shuai Sun, Baohua Li, Ren Li, Lu Liu, Xia Cui

**Affiliations:** ^1^ State Key Laboratory of Vegetable Biobreeding Sino‐Dutch Joint Laboratory of Horticultural Genomics Institute of Vegetables and Flowers Chinese Academy of Agricultural Sciences Beijing 100081 China; ^2^ College of Horticulture Northwest A&F University Yangling Shaanxi 712100 China; ^3^ Guangdong Laboratory for Lingnan Modern Agriculture State Key Laboratory for Conservation and Utilization of Subtropical Agro‐Bioresources Guangdong Basic Research Center of Excellence for Precise Breeding of Future Crops College of Agriculture South China Agricultural University Guangzhou Guangdong 510642 China; ^4^ State Key Laboratory for Crop Stress Resistance and High‐ Efficiency Production College of Horticulture Northwest A&F University Yangling Shaanxi 712100 China; ^5^ Shanghai Collaborative Innovation Center of Agri‐Seeds/Joint Center for Single Cell Biology School of Agriculture and Biology Shanghai Jiao Tong University Shanghai 200240 China; ^6^ Key Laboratory of Quality and Safety Control for Subtropical Fruit and Vegetable Ministry of Agriculture and Rural Affairs College of Horticulture Science Zhejiang A&F University Hangzhou Zhejiang 311300 China

**Keywords:** FTL2, SFT, flowering time, high temperature, tomato

## Abstract

Seasonal temperature fluctuations serve as crucial environmental cues that regulate plant reproductive timing, yet the mechanisms underlying thermoresponsive flowering regulation in tomato remain poorly understood. The study uncovers a temperature‐sensing mechanism in the tomato shoot apical meristem coordinated through antagonistic interactions between a florigen‐like protein FLOWERING LOCUS T‐Like 2 (FTL2) and florigen protein SINGLE‐FLOWER TRUSS (SFT). Under short day conditions, *FTL2* expression is specifically upregulated at the shoot apex by high temperature and functions as a flowering repressor. FTL2 physically interacts with the floral inducer SFT, directly suppressing its transcriptional activity to delay floral transition. This FTL2‐SFT regulatory circuit enables tomato precise integration of photoperiodic and thermal signals at the shoot apex, providing a molecular framework for seasonal flowering adaptation. The findings elucidate a fundamental mechanism of temperature perception in tomato flowering control and provide broader insights into how florigen‐based networks contribute to adaptive flowering control across plant species.

## Graphical abstract

This study uncovers that FLOWERING LOCUS T‐Like 2 (FTL2) and SINGLE‐FLOWER TRUSS (SFT) antagonistically regulate temperature‐responsive flowering in the tomato shoot apical meristem. High temperature induces floral repressor FTL2, which directly inhibits *SFT* to delay short‐day floral transition, while SFT reciprocally inhibits *FTL2* to promote flowering. This FTL2‐SFT module synchronizes tomato flowering with optimal environments.

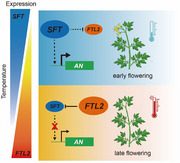

## 1 Introduction

Global warming poses a significant challenge to plant growth and development, as rising temperatures impose substantial stress on crops.^[^ ^1^, ^2^ ^]^ To cope with elevated temperatures, plants adopt two primary strategies: thermomorphogenesis, which optimizes growth patterns,^[^ ^3^ ^]^ and temperature‐sensitive flowering regulation, which ensures reproductive success. These adaptive mechanisms rely on sophisticated genetic pathways that integrate environmental signals, allowing plants to synchronize their reproductive cycles with favorable seasonal conditions – critical factors for sustaining agricultural productivity and species survival in changing climates.^[^ ^4^, ^5^ ^]^

Flowering is a finely tuned developmental transition governed by complex signaling networks integrating environmental cues, particularly photoperiod and ambient temperature.^[^ ^6^, ^7^, ^8^ ^]^ These pathways converge on key floral integrator genes, notably *FLOWERING LOCUS T* (*FT*), which encodes a mobile florigen protein. *FT* expression is induced in the leaf vasculature in response to day length, with CONSTANS acting as the primary photoperiod sensor to regulate its expression.^[^ ^9^, ^10^, ^11^, ^12^, ^13^, ^14^ ^]^ In addition to photoperiodic control, ambient temperature critically influences *FT* expression through multiple mechanisms. Temperature‐responsive regulators modulate *FT* expression to ensure flowering occurs under optimal conditions.^[^ ^15^, ^16^, ^17^, ^18^ ^]^ Moreover, temperature influences FT protein behavior at the post‐translational level, with low temperatures promoting its sequestration in phosphatidylglycerol‐enriched membranes. This restricts FT localization to companion cell membrane compartments, limiting its movement to the shoot apex.^[^ ^19^, ^20^ ^]^ Collectively, these findings highlight FT as a central hub integrating photoperiodic and temperature signals to precisely control flowering time, allowing plants to optimize reproductive timing in response to environmental fluctuations.

Plant species exhibit significant diversity in their life cycles and physiological requirements, leading to the evolution of distinct flowering strategies that align with their histories and environmental adaptation.^[^ ^21^, ^22^, ^23^, ^24^ ^]^ While tomato (*Solanum lycopersicum*) is classified as a day‐neutral plant, many cultivars and its wild relative *Solanum pimpinellifolium* tend to flower later under long‐day (LD) conditions.^[^ ^25^ ^]^ This variation in photoperiod responsiveness is primarily attributed to the function of florigen‐like genes. Two key florigen‐like regulators, *SELF PRUNING 5G* (*SP5G*), a long‐day induced flowering repressor, and *FLOWERING LOCUS T‐Like 1* (*FTL1*), a short‐day (SD) activated flowering activator, collectively regulate *SELF PRUNING* (*SFT*) expression, the tomato ortholog of *FT*.^[^ ^25^, ^26^, ^27^ ^]^ Allelic variations in *SP5G* and *FTL1* that confer insensitivity to photoperiod were selected during domestication, facilitating the widespread cultivation of day‐neutral tomato.^[^ ^27^ ^]^

Beyond the photoperiod, ambient temperature exerts a profound influence on tomato development, affecting key processes from flowering to fruit set and ripening. Elevated temperatures, particularly during the reproductive phase, can severely disrupt pollen viability, impair fertilization, and reduce fruit set, ultimately diminishing yields.^[^ ^28^, ^29^, ^30^ ^]^ Despite its agricultural significance, the molecular mechanisms governing temperature‐dependent flowering regulation in tomato remain poorly understood. Our study reveals that *FTL2*, a florigen‐like gene, is specifically induced by high temperature in the shoot apex under short‐day conditions, where it acts as a potent floral repressor. Once activated, *FTL2* directly inhibits the function of *SFT*, a key florigen gene required for flowering. By upregulating *FTL2* while repressing *SFT*, high temperature effectively delays the floral transition. This *FTL2*‐*SFT* antagonism fine‐tunes flowering time by integrating temperature and photoperiodic cues, ensuring a balance between vegetative growth and reproductive development. Our results highlight the crucial role of the *FTL2*‐*SFT* regulatory network in high‐temperature‐mediated flowering in tomato, providing insights into how tomato adapt their reproductive timing to environmental fluctuations and offering potential strategies to enhance crop resilience in a changing climate.

## 2 Results

### 2.1 High Temperature Represses Tomato Flowering Under SD Conditions

**Figure 1:**
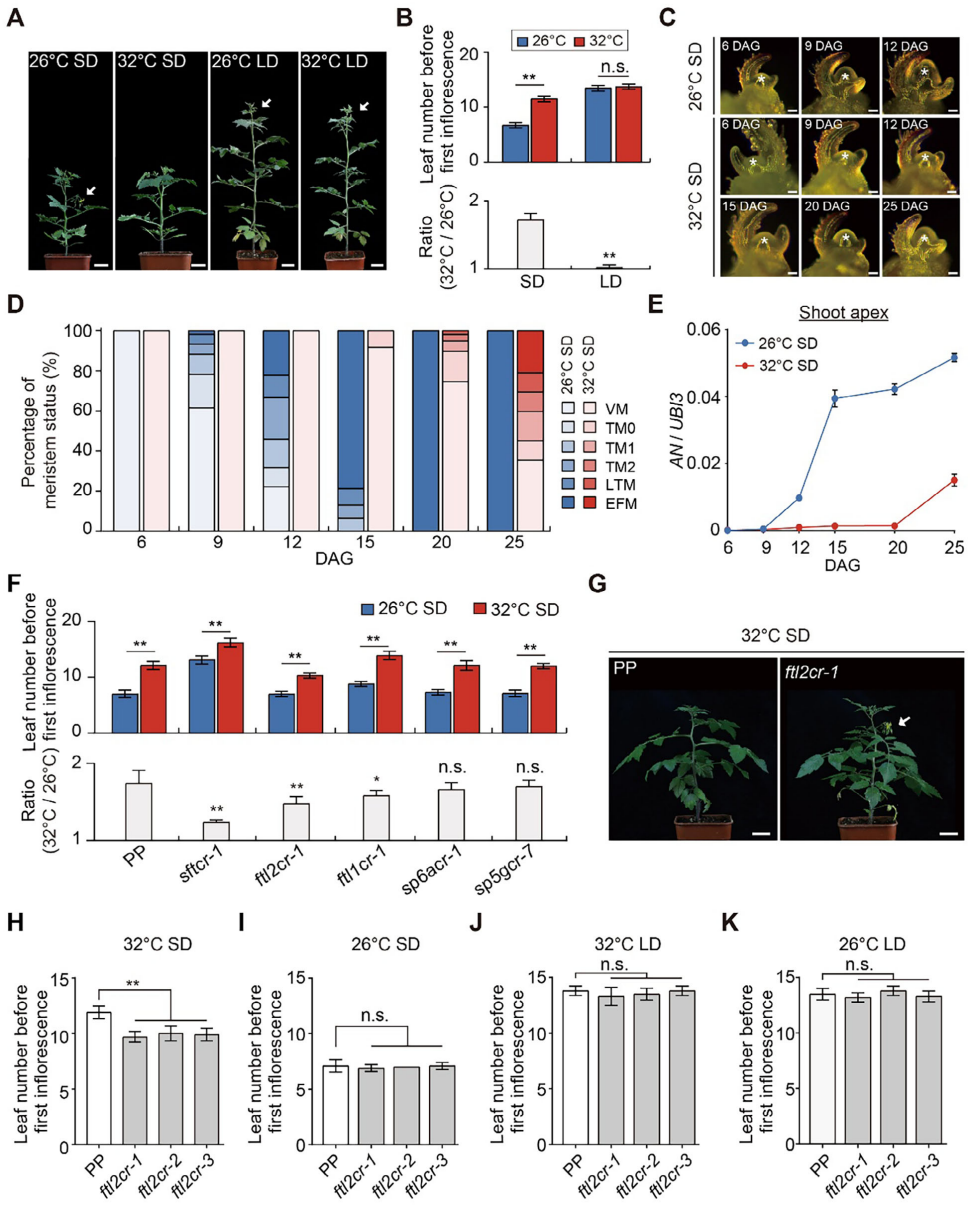
*FTL2* represses flowering at high temperatures under SD conditions. A) Phenotypes of PP grown at 26 and 32 °C under short‐day (SD) and long‐day (LD) conditions. White arrowheads indicate primary inflorescences. Scale bar: 3 cm. B) Leaf number before the first inflorescence in PP grown at 26 and 32 °C under SD and LD conditions (upper panels). The ratios of leaf number between 32 and 26 °C (32/26 °C) for PP is shown in the lower panel. Data are mean ± s.d. (*n* = 10–14). Data were compared by two‐tailed Student's *t*‐test, ^**^ *p* <0.01; n.s., no significance (*p* >0.05). C) Representative images of the shoot apical meristem during floral transition at 26 and 32 °C under SD conditions. The asterisks indicate the position of the shoot apical meristem. DAG: Days after germination. Scale bar: 50 µm. D) Percentage of meristem status at 6, 9, 12, 15, 20, and 25 DAG under 26 °C‐ and 32 °C‐SD conditions (*n* = 55–65). Meristem maturation is characterized by six sequential stages of primary shoot meristem development: VM (vegetative meristem), TM0 (transition meristem), TM1, TM2, LTM (late‐transition meristem), and EFM (early‐flowering meristem). E) Relative temporal expression of *AN* in the shoot apical meristem (SAM) of developing PP seedlings at 26 and 32 °C under SD conditions. The expression levels were normalized to *UBI3*. Data are presented as mean ± s.d. (*n*  =  3). F) Leaf number before the first inflorescence in PP, *sftcr‐1*, *ftl2cr‐1*, *ftl1cr‐1*, *sp6acr‐1*, and *sp5gcr‐7* grown at 26 and 32 °C under SD conditions (upper panels). The ratios of leaf number between 32 and 26 °C (32/26 °C) for all genotypes are shown in the lower panel. Data are mean ± s.d. (*n* = 10). Data were compared by two‐tailed Student's *t*‐test, ^**^ *p* <0.01; ^*^ *p* <0.05; n.s., no significance (*p* >0.05). G) Wild‐type and *ftl2cr‐1* plants showing earlier flowering of the primary shoot in *ftl2cr‐1*. White arrowhead marks an open primary inflorescence at anthesis in a *ftl2cr‐1* mutant. Scale bar: 3 cm. H–K) Leaf number before the first inflorescence in PP and *ftl2cr* mutants grown under 32 °C‐SD (H), 26 °C‐SD (I), 32 °C‐LD (J), and 26 °C‐LD (K) conditions. Data are mean ± s.d. (*n* = 10). Data were compared by two‐tailed Student's *t*‐test, ^**^ *p* <0.01; n.s., no significance (*p* >0.05).

The wild progenitor of cultivated tomato, *Solanum pimpinellifolium* (PI365967, PP), is a facultative SD plant.^[^ ^27^ ^]^ Its flowering is significantly delayed when grown under LD conditions (**Figure** **1**A,B). To investigate how ambient temperature affects tomato flowering, we examined the flowering time of PP at two different temperatures (26 and 32 °C) under both LD and SD conditions. High temperature (32 °C) had a pronounced effect on the flowering of wild tomato plants under SD conditions. Under these conditions, flowering was significantly delayed, as evidenced by an increase in the number of leaves produced before the first inflorescence emerged. However, under LD conditions, the impact of high temperature on flowering was minimal (Figure 1A,B). This differential response highlights that wild tomato plants' sensitivity to ambient temperature changes is highly dependent on the photoperiod.

During the floral transition, the shoot apical meristem (SAM) progresses from the vegetative meristem (VM) to the early‐flowering meristem (EFM) through six distinct morphological phases.^[^ ^31^ ^]^ We found that elevated temperature significantly delays this transition under SD conditions. Specifically, the late‐transition meristem (LTM) typically emerged by 12 days after germination (DAG) at 26 °C, whereas at 32 °C conditions, LTM appearance was delayed until ≈25 DAG (Figure 1C,D). However, this temperature‐dependent delay in reproductive transition was not observed under LD conditions (Figure S1, Supporting Information), indicating that photoperiod regulates thermal responses in tomato flowering regulation.

In tomato, the *ANANTHA* (*AN*) is a floral meristem identity gene that plays a crucial role in flower formation.^[^ ^32^, ^33^, ^34^ ^]^ Spatial expression profiling confirmed that *AN* is specifically expressed in the floral meristem (FM) (Figure S2A,B, Supporting Information). To investigate the thermal regulation of floral transition, we examined *AN* expression dynamics at 26 and 32 °C under SD conditions. We found that the upregulation of *AN* was significantly delayed at 32 °C compared to 26 °C (Figure 1E), temporally correlating with the observed developmental delay in SAM transition. Importantly, this temperature‐induced delay in gene induction did not occur under LD conditions (Figure S2C, Supporting Information), further supporting that temperature‐induced changes in the SAM transition are modulated by photoperiod.

### 2.2 FTL2 Specifically Represses Flowering Under High Temperatures

FT serves as a central integrator of ambient temperature signals to regulate flowering time in *Arabidopsis*.^[^ ^15^, ^16^, ^17^ ^]^ Across plant species, the *FT* gene family has undergone duplication and significant functional diversification, leading to the evolution of distinct regulatory roles.^[^ ^35^, ^36^ ^]^ Florigen and florigen‐like genes are expressed in leaves and shoot apical meristems, where they play crucial roles in regulating the reproductive transitions of plants.^[^ ^26^, ^37^ ^]^ In tomato, five phylogenetically distinct *FT*‐like paralogs have been identified: *SFT*, *FTL1*, *FTL2*, *SP5G*, and *SELF PRUNING 6A* (*SP6A*) (Figure S3, Supporting Information). To elucidate their roles in thermoresponsive flowering, we analyzed their spatial and thermal expression patterns in leaves, VM, and transition meristem (TM) under 26 °C‐ and 32 °C‐SD conditions (Figure S4, Supporting Information). Our analysis revealed that *SP5G* and *FTL1* exhibited predominant expression in leaves, consistent with their roles in photoperiod‐responsive flowering regulation. Notably, we observed that *SFT* and *FTL2* were primarily expressed in TMs but exhibited opposing responses to high temperature. While *SFT* was highly expressed in TMs under normal conditions, its expression was significantly repressed at elevated temperatures. In contrast, *FTL2* was strongly upregulated in meristem tissues under 32 °C‐SD conditions, suggesting its involvement in high‐temperature sensing and flowering time regulation (Figure S4, Supporting Information). These findings indicate a functional divergence among *FT*‐like paralogs, with distinct roles in integrating temperature signals to fine‐tune flowering.

To explore the functions of these *FT‐*like genes in regulating flowering in responsive to high temperature, we systematically evaluated flowering phenotypes of *sftcr‐1*, *ftl1cr‐1*, *ftl2cr‐1*, *sp5gcr‐7*, and *sp6acr‐1*, which were previously or newly generated CRISPR‐edited plants,^[^ ^27^ ^]^ under 26 °C‐ and 32 °C‐SD conditions (Figure S5A, Supporting Information). The *sftcr‐1*, *ftl1cr‐1*, and *ftl2cr‐1* mutants exhibited varying degrees of thermal insensitivity. Both s*ftcr‐1* and *ftl1cr‐1* mutants showed delayed flowering at both temperatures (Figure 1F). However, the *ftl2cr‐1* mutant uniquely accelerated flowering specifically under 32 °C‐SD conditions (Figure 1F,G). This consistent phenotypic response was confirmed across multiple *ftl2cr* mutant alleles (Figure 1H–K), indicating that FTL2 functions as a high‐temperature‐specific floral repressor.

Evolutionary analysis identified key residues that distinguish floral inducers from repressors in FT homologs.^[^ ^35^, ^38^, ^39^ ^]^ While floral activators like *Arabidopsis* FT are characterized by conserved tyrosin (Y134) and tryptophan (W138) residues, FTL2 undergoes an amino acid substitution at position 138, replacing tryptophan with asparagine (W138N) (Figure S5B, Supporting Information). This alteration underlies its ability to function as a floral repressor. Collectively, these findings highlight the critical role of FTL2 in temperature‐dependent flowering repression.

### 2.3 *FTL2* Expression is Induced in the Shoot Apex by High‐Temperature

**Figure 2:**
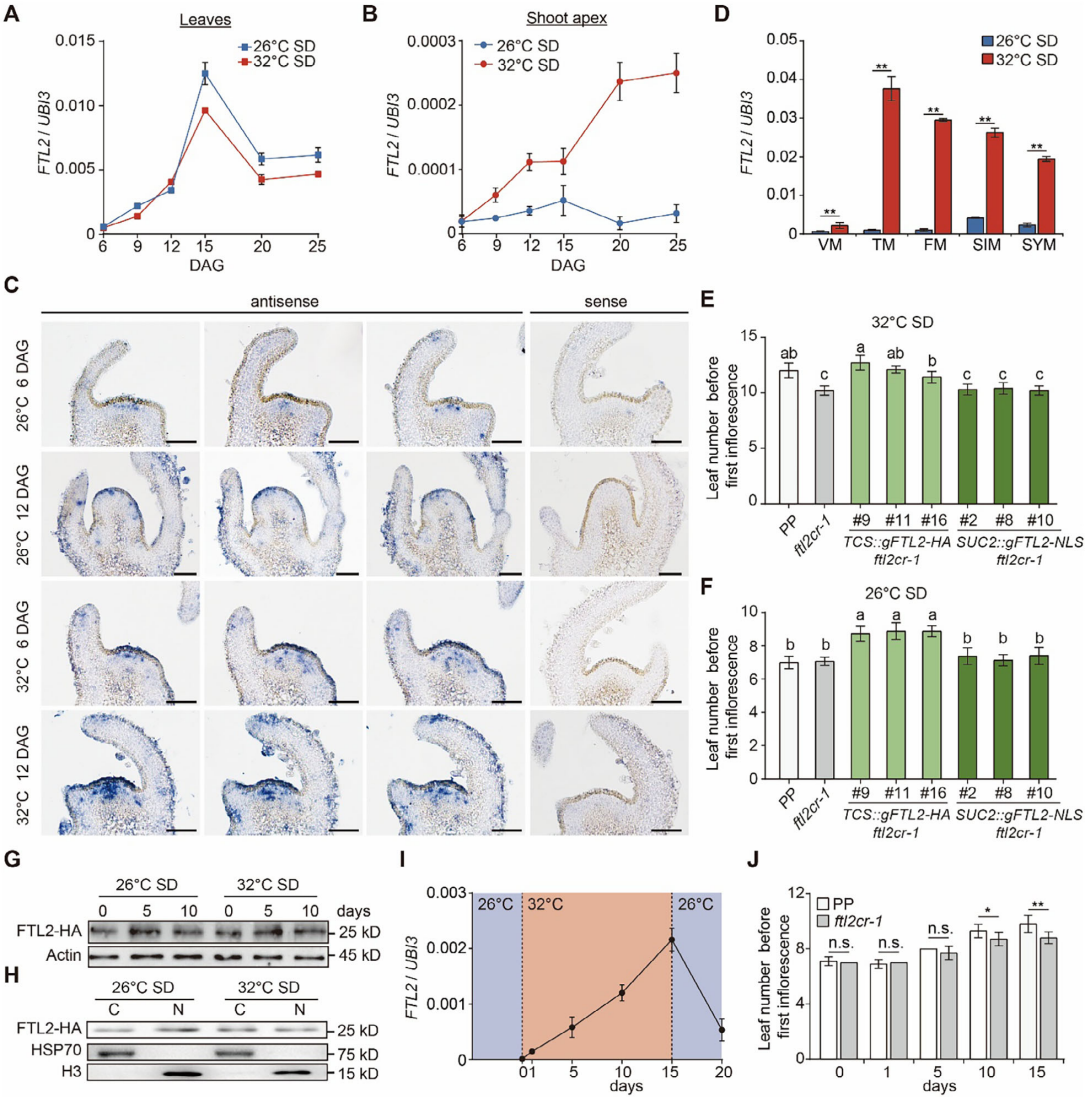
High temperature induces *FTL2* expression in the shoot apex. A,B) Temporal expression profiles of *FTL2* in leaves (A) and shoot apical meristems (B) of developing PP seedlings at 26 and 32 °C under SD conditions. Expression levels were normalized to *UBI3*. Data are presented as mean ± s.d. (*n*  =  3). C) In situ hybridization of *FTL2* in wild‐type PP shoot apical meristems at 6 DAG and 12 DAG under 26 °C‐ and 32 °C‐SD conditions. Scale bar: 50 µm. D) qRT‐PCR analysis of *FTL2* expression in different meristematic tissues of PP at 26 and 32 °C under SD conditions. Expression levels were normalized to *UBI3*. Data are presented as mean ± s.d. (*n*  =  3). Data were compared by a two‐tailed Student's *t*‐test, ^**^ *p* <0.01. E,F) Leaf number before the first inflorescence in *TCS::gFTL2‐HA ftl2cr‐1* and *SUC2::gFTL2‐NLS ftl2cr‐1* transgenic plants grown under 26 °C‐SD (E) and 32 °C‐SD (F) conditions. Data are presented as mean ± s.d. (*n*  = 10–16). Different letters denote significant differences (*p* <0.05) based on one‐way ANOVA followed by Tukey's multiple comparisons test. G) Western blot analysis of FTL2‐HA protein levels in shoot apical meristems of *TCS::gFTL2‐HA ftl2cr‐1* during the transition from 26 to 32 °C using anti‐HA antibody (Sigma; H6908). Seedlings were grown for 5 DAG at 26 °C, then transferred to 32 °C for 0, 5, and 15 days. Control seedlings were maintained at 26 °C. Samples were collected and analyzed at the indicated time points. Anti‐actin antibody (Sigma; A0480) was used as the control. H) Abundance of FTL2 protein in the cytoplasm and nucleus in shoot apical meristems of *TCS::gFTL2‐HA ftl2cr‐1* at 26 and 32 °C under SD conditions. Anti‐HSP70 and anti‐H3 antibodies were used as cytoplasmic and nuclear markers, respectively. C, cytoplasm; N, nucleus. I) Relative temporal expression of *FTL2* in shoot apical meristems of PP after temperature shifts. Seedlings were grown for 5 DAG at 26 °C, then transferred to 32 °C for 0, 1, 5, 10, and 15 days, and subsequently returned to 26 °C for an additional 5 days. Samples were collected and analyzed at the specified time points. Expression levels were normalized to *UBI3*. Data are presented as mean ± s.d. (*n*  =  3). J) Leaf number before the first inflorescence in PP and *ftl2cr‐1* mutants after transferring 5 DAG seedlings from 26 to 32 °C for 0, 1, 5, 10, and 15 days. Data are presented as mean ± s.d. (*n*  =  10–11) Data were compared by two‐tailed Student's *t*‐test, ^**^ *p* <0.01; ^*^ *p* <0.05; n.s., no significance (*p* >0.05).

Since FTL2 regulates flowering specifically under 32 °C‐SD conditions, we detected its temperature‐specific activity. We first examined *FTL2* expression dynamics in leaves and SAMs. During the floral transition, *FTL2* expression in leaves remained unaffected by temperature differences between 26 and 32 °C under SD conditions (**Figure** **2**A). However, *FTL2* expression was significantly induced in the SAM at high temperatures (Figure 2B). In situ hybridization confirmed this spatial specificity, showing intensified *FTL2* mRNA signals exclusively in SAMs at elevated temperatures (Figure 2C). To explore this further, we examined *FTL2* expression across various meristem tissues, including VM, TM, FM, sympodial inflorescence meristem (SIM), and sympodial shoot meristem (SYM). The results revealed that its thermal induction occurred in meristematic regions, whereas no temperature‐induced *FTL2* expression was detected in leaves or the shoot apex under LD conditions (Figure 2D; Figure S6, Supporting Information). These results suggest that *FTL2* induction in SAMs serves as a hallmark of thermoresponsive flowering regulation in tomato.

To determine whether FTL2 regulates flowering at the shoot apex, we performed tissue‐targeted complementation in *ftl2cr‐1* mutants. The *TCS::gFTL2‐HA* transgenic lines, in which *FTL2* was expressed in the SAM under the control of the *TCS* promoter,^[^ ^31^, ^40^ ^]^ fully rescued the early flowering phenotype of *ftl2cr‐1* at 32 °C. In contrast, *SUCROSE TRANSPORTER 2* (*SUC2*)*::gFTL2‐NLS*, which drives higher *FTL2* expression in phloem companion cells,^[^ ^41^ ^]^ failed to restore the mutant phenotype (Figure 2E; Figure S7, Supporting Information). At 26 °C, overexpression of *FTL2* in SAM further delayed flowering, while phloem‐specific expression had no effect (Figure 2F). These results demonstrate that FTL2 exerts its flowering repression function within the SAM.

To determine whether FTL2 transcripts or proteins are directly influenced by ambient temperature, we compared FTL2‐HA mRNA and protein levels in the shoot apices of *TCS::gFTL2‐HA ftl2cr‐1* transgenic plants continuously grown at 26 °C with those transferred to 32 °C under SD conditions. Neither FTL2‐HA transcript abundance nor protein stability differed between 26 and 32 °C in shoot apices (Figure 2G; Figure S8A, Supporting Information), ruling out temperature‐dependent mRNA or protein turnover. Additionally, subcellular fractionation analysis confirmed that FTL2‐HA maintained a consistent nuclear‐cytoplasmic partitioning across temperatures (Figure 2H), a pattern also observed in transiently transformed *35S::FTL2‐eGFP* tobacco epidermal cells (Figure S8B, Supporting Information). These results collectively indicate that FTL2 protein levels and subcellular localization are not directly regulated by ambient temperature.

To elucidate the temperature sensitivity of FTL2, we subjected plants to a controlled thermal shift, exposing them to 32 °C for 15 days followed by re‐acclimation to 26 °C under SD conditions. We observed a progressive induction of *FTL2* expression during heat exposure, followed by a sharp decline upon reversion to 26 °C (Figure 2I). This transcriptional switching highlights the role of FTL2 as a dynamic rheostat for integrating thermal inputs. Phenotypic analysis revealed that prolonged exposure to 32 °C exacerbated the early flowering phenotype in *ftl2cr‐1* mutants (Figure 2J), establishing a direct correlation between thermal exposure duration and flowering acceleration. These findings collectively establish FTL2 as a SAM‐localized transcriptional integrator that converts thermal exposure duration into precise developmental timing.

### 2.4 FTL2 Represses *SFT* Expression in the Shoot Apex at Elevated Temperatures

**Figure 3:**
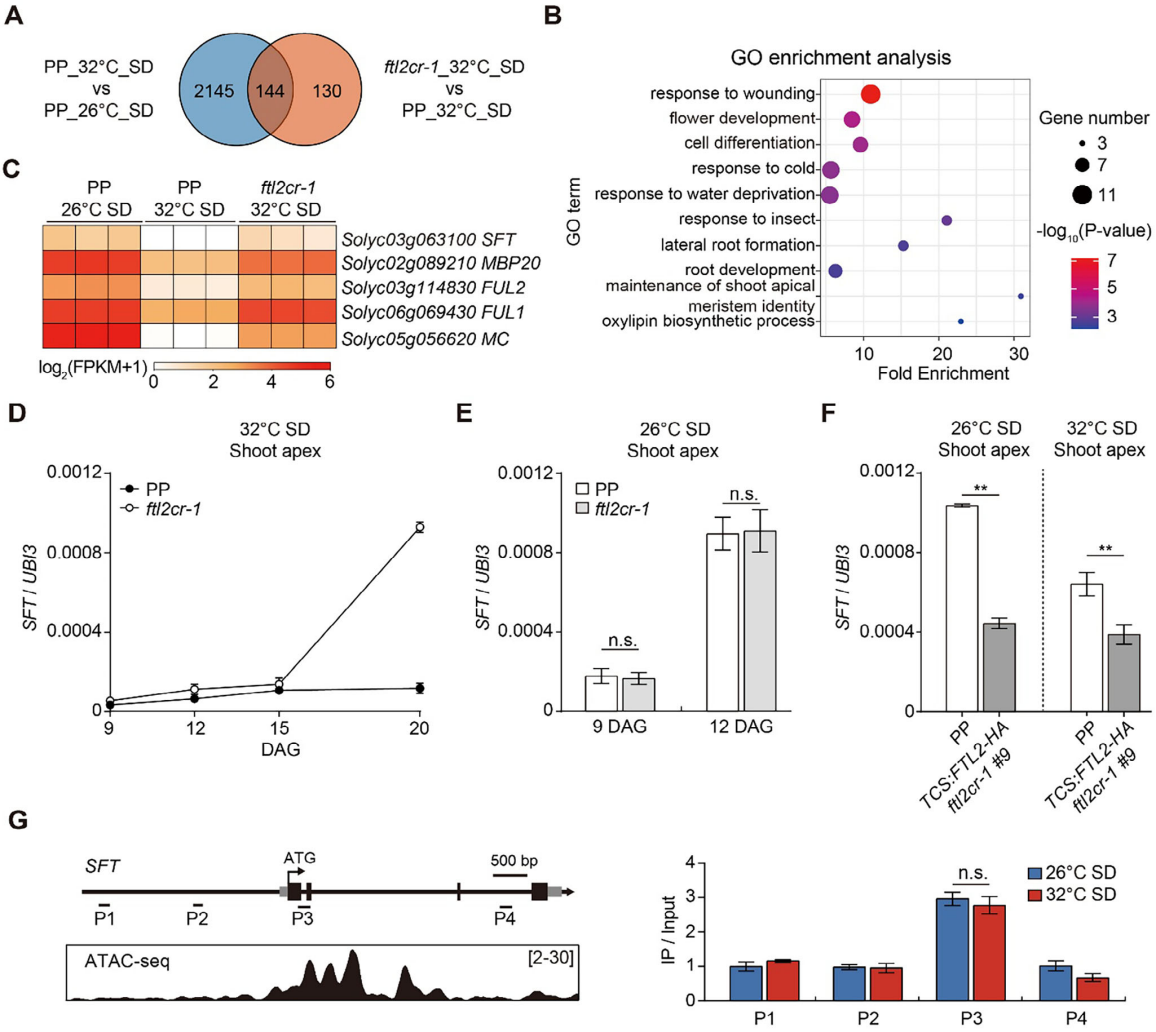
FTL2 represses *SFT* expression in the shoot apex at high temperatures under SD conditions. A) Venn diagram showing the number of overlapping genes between differentially expressed genes in PP_26 °C_SD relative to PP_32 °C_SD and differentially expressed genes in PP_32 °C_SD relative to *ftl2cr‐1*_32 °C_SD as identified by RNA‐seq. B) Gene ontology (GO) term enrichment analysis of overlapping genes. The top ten most significantly enriched GO terms in the biological process category, ranked by *p*‐values derived from a statistical over‐representation test, are displayed. C) Heatmaps depicting the expression profiles of key flowering time genes in terms of flower development. D) Temporal expression profiles of *SFT* in the shoot apex of developing PP and *ftl2cr‐1* seedlings under 32 °C‐SD conditions. E) Relative *SFT* expression level in shoot apical meristems of PP and *ftl2cr‐1* plants at 9 DAG and 12 DAG under 26 °C‐SD conditions. F) Relative *SFT* expression level in the shoot apical meristems of PP and *TCS::gFTL2‐HA ftl2cr‐1 #9* plants at 12 DAG under 26 °C‐SD conditions and at 25 DAG under 32 °C‐SD conditions. Expression levels in D, E, and F were normalized to *UBI3*. Data are presented as mean ± s.d. (*n*  =  3). Data were compared by two‐tailed Student's *t*‐test, ^**^ *p* <0.01; n.s., not significant (*p* >0.05). G) ChIP‐qPCR assays demonstrating FTL2 enrichment on the genomic DNA of *SFT*. Upper left: Schematic representation of the *SFT* gene, with transverse lines indicating regions analyzed by ChIP‐qPCR. The translation start site (ATG) is denoted at position +1. Lower left: Meristem ATAC‐seq coverage is displayed. Data were obtained from Hendelman et al. (2021). Input DNA served as the negative control, and the intergenic region near *ACTIN* (*Solyc03g078400*) was used as an internal control. Data are presented as mean ± s.d. (*n*  =  3). Data were compared by two‐tailed Student's *t*‐test, n.s., no significance (*p* >0.05).

To explore the roles of FTL2 in thermosensitive flowering, we performed a genome‐wide transcriptomic profiling of shoot apices of PP and *ftl2cr‐1* plants grown under 26 °C‐ and 32 °C‐SD conditions. Comparative analysis with a 1.5‐fold cutoff revealed 2289 differentially expressed genes (DEGs) between 26 °C‐ and 32 °C‐grown wild‐type PP plants (Table S1, Supporting Information), with 144 overlapping genes (52.6%) co‐regulated by FTL2 under high temperature (**Figure** **3**A; Table S2, Supporting Information). This substantial overlap demonstrates that FTL2 is a key regulator of temperature‐mediated flowering time control. Gene Ontology (GO) enrichment highlighted the roles of these genes in floral development, maintenance of shoot apical meristem identity, and cell differentiation (Figure 3B), suggesting FTL2 as a thermal integrator in SAM reprogramming.

The FT ortholog SFT acts as the florigen to promote tomato flowering.^[^ ^42^, ^43^ ^]^ RNA‐seq revealed that *SFT* expression was significantly suppressed under 32 °C‐SD conditions compared to under 26 °C‐SD conditions, aligning with the delayed flowering observed in wild‐type PP (Figure 1A). However, this thermal repression was partially alleviated in *ftl2cr‐1* mutants (Figure 3C), identifying FTL2 as a key regulator of *SFT* downregulation. Spatial expression analysis via qRT‐PCR confirmed that *SFT* levels were significantly elevated in the shoot apices of *ftl2cr‐1* seedlings compared to PP, but only at 32 °C (Figure 3D,E). Complementation *ftl2cr‐1* with *TCS::gFTL2‐HA* restored *SFT* repression and delayed flowering (Figures 2E,F and 3F). These reciprocal genetic perturbations establish FTL2 as a thermoresponsive transcriptional repressor of *SFT* in the shoot apex, emphasizing its role in meristem‐localized temperature modulation of floral timing in tomato.

To determine whether FTL2 directly regulates *SFT* transcription, we performed chromatin immunoprecipitation (ChIP) assays in the *TCS::gFTL2‐HA ftl2cr‐1* transgenic plants under 26 °C‐ and 32 °C‐SD conditions. Significant enrichment FTL2‐HA was detected at the P3 region (spanning exons 1–2 of *SFT*) (Figure 3G), a locus previously identified as an open chromatin domain via Assay for Transposase‐Accessible Chromatin using sequencing (ATAC‐seq).^[^ ^44^ ^]^ These results supported the idea that FTL2 binds *SFT* in vivo to regulate its transcription in SAM. However, the association of FTL2 with the *SFT* promoter did not change significantly between 26 and 32 °C (Figure 3G), indicating that the binding affinity of FTL2 to *SFT* remains unchanged regardless of ambient temperature.

Florigen interacts with 14‐3‐3 scaffolding proteins and a basic region/leucine zipper transcription factor to assemble the florigen activation complex (FAC), a crucial regulatory module that activates the downstream floral meristem identity genes.^[^ ^45^ ^]^ In tomato, the flowering regulators SFT and FTL1 have been demonstrated to recruit 14‐3‐3 proteins to promote the transcriptional activation of flowering‐related genes.^[^ ^27^, ^46^ ^]^ To investigate whether FTL2 shares a conserved mechanism with these FT‐like proteins, we systematically analyzed its interaction with all 14‐3‐3 isoforms. No detectable binding was observed between FTL2 and any 14‐3‐3 family member in yeast two‐hybrid assays (Figure S9A, Supporting Information). Furthermore, FTL2 failed to interact with SPGB (SP‐interacting G‐BOX), an FD‐like transcription factor critical for the FAC complex (Figure S9B, Supporting Information).

Structural analysis of the rice Hd3a‐GF14c‐OsFD1 complex has previously delineated conserved binding interfaces between Hd3a (a florigen homolog) and 14‐3‐3 proteins.^[^ ^45^ ^]^ Comparative sequence alignment of FT‐like proteins across species revealed that these interaction motifs are evolutionarily conserved but harbor distinct substitutions in FTL2 (F63, Q64, and S98). To investigate whether these mutations in FTL2 disrupt its ability to interact with 14‐3‐3 proteins, we generated a mutant protein (FTL2^Mu^, F63L/Q64R/S98T) that restores the consensus sequence found in canonical FT proteins (Figure S9C, Supporting Information). Interestingly, the mutant protein FTL2^Mu^ regained the ability to interact with 14‐3‐3 proteins, yet it remained incapable of binding SPGB (Figure S9A,B, Supporting Information). These findings suggest that FTL2 regulates flowering through a non‐canonical pathway independent of the classical FAC complex.

### 2.5 Spatio‐Temporal Regulation of *SFT* Expression

**Figure 4:**
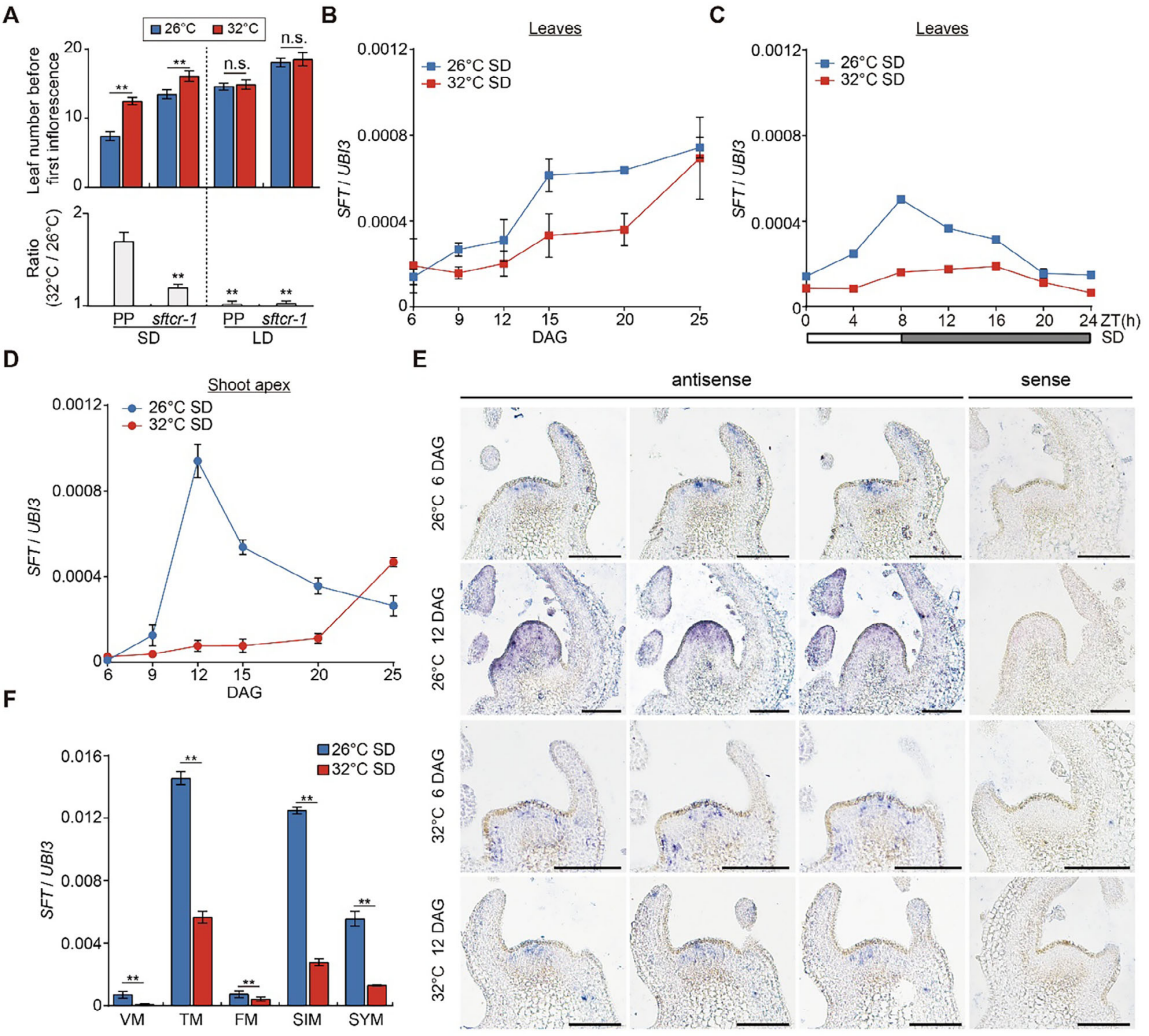
High temperature represses *SFT* expression in the shoot apex under SD conditions. A) Leaf number before the first inflorescence in PP and the *sftcr‐1* mutant grown at 26 and 32 °C under SD and LD conditions are shown in the upper panels. The ratios of leaf number between 32 and 26 °C (32/26 °C) for PP and the *sftcr‐1* mutant are presented in the lower panel. Data are presented as mean ± s.d. (*n*  =  13–14). Data were compared by two‐tailed Student's *t*‐test, ^**^ *p* <0.01; n.s., no significance (*p* >0.05). B) Relative temporal expression levels of *SFT* in leaves of developing PP seedlings at 26 and 32 °C under SD conditions. C) Diurnal oscillation of *SFT* in 12‐day‐old PP seedlings at 26 and 32 °C under SD conditions. Sampling time is expressed in hours as ZT (zeitgeber time), representing the number of hours after the onset of illumination. D) Relative temporal expression levels of *SFT* in the shoot apex of developing PP seedlings at 26 and 32 °C under SD conditions. E) In situ hybridization of *SFT* in wild‐type PP shoot apical meristems at 6 DAG and 12 DAG under 26 °C‐ and 32 °C‐SD conditions. Scale bar: 50 µm. F) qRT‐PCR analysis of the relative expression of *SFT* in different meristematic tissues of PP at 26 and 32 °C under SD conditions. Expression levels in B, C, D, and F were normalized to *UBI3*. Data are presented as mean ± s.d. (*n*  =  3). Data were compared by a two‐tailed Student's *t*‐test, ^**^ *p* <0.01.

The FT ortholog SFT regulates the primary shoot flowering time in tomato.^[^ ^42^, ^43^ ^]^ The *sftcr‐1* mutants exhibited delayed flowering under both LD and SD conditions compared to wild‐type plants.^[^ ^42^ ^]^ We found that the high‐temperature (32 °C)‐inhibited flowering was significantly attenuated in *sftcr‐1* under SD conditions (Figure 1F; **Figure** **4**A). This partial suppression of thermal responsiveness in the *sftcr‐1* mutant implicates SFT in mediating temperature‐dependent flowering regulation in tomato.

Although *SFT* is repressed by FTL2 in SAM, it remains unclear whether *SFT* expression levels in the SAM are essential for high‐temperature flowering in tomato. To address this, we examined *SFT* expression in developing PP seedlings under different conditions. The results showed that *SFT* expression was gradually increased in leaves from vegetative to reproductive phases at both 26 and 32 °C, albeit with modest thermal suppression at elevated temperatures (Figure 4B). As *SFT* exhibits a diurnal expression pattern under SD conditions, with a peak at ZT8,^[^ ^27^, ^47^ ^]^ we also measured its circadian expression every 4 h over a 24‐h period under 26 °C‐ and 32 °C‐SD conditions. *SFT* expression was persistently suppressed at 32 °C throughout the photoperiod (Figure 4C).

Different with that *SFT* expression in leaves was only mildly suppressed under high temperature, its mRNA levels in the shoot apex showed significant divergence between temperatures: a pronounced pre‐floral transcriptional peak observed at 12 DAG under 26 °C‐SD conditions was absent under 32 °C‐SD conditions (Figure 4D). Spatiotemporal analysis using in situ hybridization demonstrated that *SFT* mRNA specifically accumulated in the SAM during the floral transition under 26 °C‐SD conditions; however, this SAM‐localized expression pattern was strikingly absent under 32 °C‐SD conditions (Figure 4E). *SFT* expression analysis across meristematic tissues revealed that *SFT* was strongly expressed in all these meristem tissues under 26 °C. Still, its expression was consistently suppressed at 32 °C (Figure 4F). Notably, this thermal repression of *SFT* expression at high temperatures was absent under LD conditions (Figure S10, Supporting Information). These findings highlight that high temperatures repress *SFT* expression, with the most pronounced suppression occurring in the SAM, thereby impacting the thermoresponsive floral transition in tomato.

### 2.6 *SFT* Induction in Shoot Apex Mediates Thermoresponsiveness

**Figure 5:**
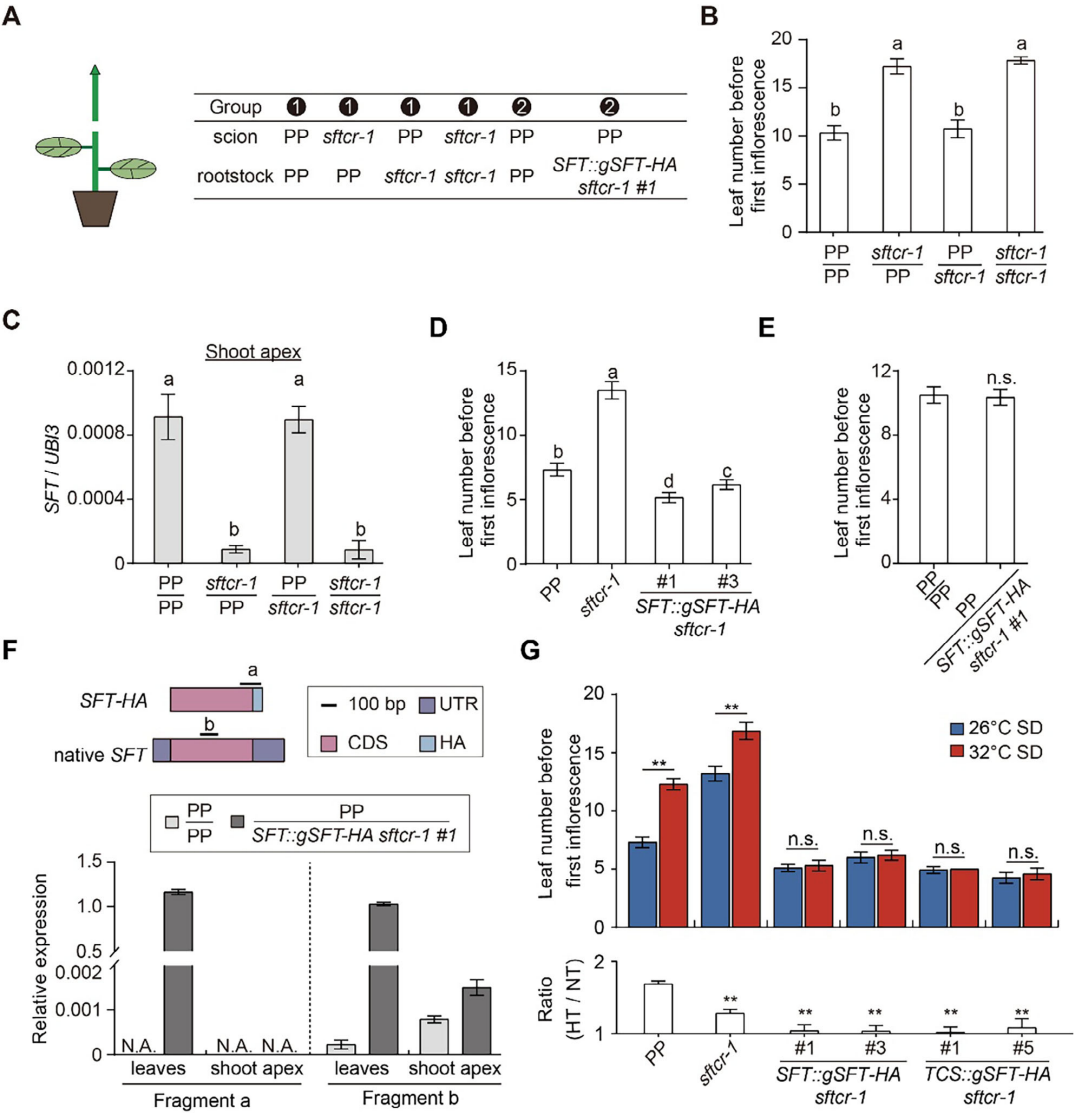
Shoot apex *SFT* induction mediates thermoresponsiveness in plants. A) On the left, a schematic diagram depicts the grafting procedure. The table on the right summarizes the scion and rootstock combinations used in two sets of grafting experiments. B) Leaf number before the first inflorescence in grafted plants: PP or *sftcr‐1* scions on PP or *sftcr‐1* rootstocks. Data are presented as mean ± s.d. (*n*  =  15–20). C) Relative expression levels of *SFT* in the shoot apical meristems of scions: PP or *sftcr‐1* scions on PP or *sftcr‐1* rootstocks. Expression levels were normalized to *UBI3*. Data are presented as mean ± s.d. (*n*  =  3). D) Leaf number before the first inflorescence in PP, *sftcr‐1*, and *SFT::gSFT‐HA sftcr‐1* plants grown at 26 °C under SD conditions. Data are presented as mean ± s.d. (*n*  =  14). Different lowercase letters in B, C, and D denote statistically significant differences (*p* <0.05), as determined by one‐way ANOVA followed by Tukey's multiple comparisons test. E) Leaf number before the first inflorescence in grafted plants: PP scions on PP or *SFT::gSFT‐HA sftcr‐1 #1* rootstocks. Data are presented as mean ± s.d. (*n*  =  16–19). Data were compared by two‐tailed Student's *t*‐test, n.s., not significant (*p* >0.05). F) Upper: Schematic diagrams depicting *SFT‐HA* transcripts of *SFT::gSFT‐HA sftcr‐1* and native *SFT* transcripts. Fragments labeled as “a” and “b” represent amplicons analyzed in qRT‐PCR assays. Lower: Relative expression levels of *SFT‐HA* and *SFT* in leaves of rootstocks and shoot apical meristems of scions: PP scions on PP or *SFT::gSFT‐HA sftcr‐1 #1* rootstocks. Expression levels were normalized to *UBI3*, and data are presented as mean ± s.d. (*n*  =  3). G) Leaf number before the first inflorescence in PP, *sftcr‐1*, *SFT::gSFT‐HA sftcr‐1*, and *TCS::gSFT‐HA sftcr‐1* plants grown at 26 and 32 °C under SD conditions (upper panels). The ratios of leaf number between 32 and 26 °C (32/26 °C) for all genotypes are displayed in the lower panel. Data are presented as mean ± s.d. (*n*  =  10–12). Data were compared by two‐tailed Student's *t*‐test, ^**^ *p* <0.01; n.s., no significance (*p* >0.05).

SFT functions as a leaf‐derived mobile florigenic signal in tomato.^[^ ^43^ ^]^ However, the reciprocal grafting experiments demonstrated that wild‐type PP donors could not rescue the delayed flowering phenotype of *sftcr‐1* mutants. By contrast, PP scions flowered normally irrespective of the rootstock genotype (**Figure** **5**A,B). These findings suggested that meristem‐autonomous *SFT* induction is essential for floral commitment. Consistent with this, SAM‐localized *SFT* expression was detected exclusively in grafts with PP scions (Figure 5C). Collectively, these findings demonstrate that autonomous SFT activity in the shoot apex, along with mobile leaf‐derived signals, is critical for promoting floral transition.

Given recent evidence for *FT* mRNA mobility,^[^ ^48^, ^49^, ^50^ ^]^ we tested whether the mobility of *SFT* transcript contributes to the increased *SFT* levels in the shoot apex. The transgenic *SFT::gSFT‐HA sftcr‐1* lines, which overexpress functional *SFT‐HA*, fully rescued the phenotypes of the *sftcr‐1* mutant (Figure 5D; Figure S11A,B, Supporting Information). However, grafting wild‐type scions onto these rootstocks did not elevate *SFT* mRNA in the SAM or accelerate flowering, and no *SFT‐HA* transcripts were detected in the scions (Figure 5E,F). This indicates that intrinsic transcriptional activation in SAM, rather than mRNA trafficking, is responsible for *SFT* accumulation in the apex. Furthermore, we found that ectopic expression of *SFT* in *SFT::gSFT‐HA sftcr‐1* lines significantly alleviated the high‐temperature‐induced delay in flowering (Figure 5G). To further explore the role of *SFT* expression in the SAM, we generated *TCS::gSFT‐HA sftcr‐1* transgenic lines in which *SFT* was expressed in the SAM under the control of the *TCS* promoter (Figure S11C, Supporting Information). Notably, *TCS::gSFT‐HA sftcr‐1* transgenic lines exhibited temperature‐insensitive early flowering, which was attributed to elevated *SFT* levels in the SAM (Figure 5G). Together, these findings establish a direct link between *SFT* levels in the meristem and thermoresponsiveness in tomato.

### 2.7 SFT Represses *FTL2* Expression in the Shoot Apex

**Figure 6:**
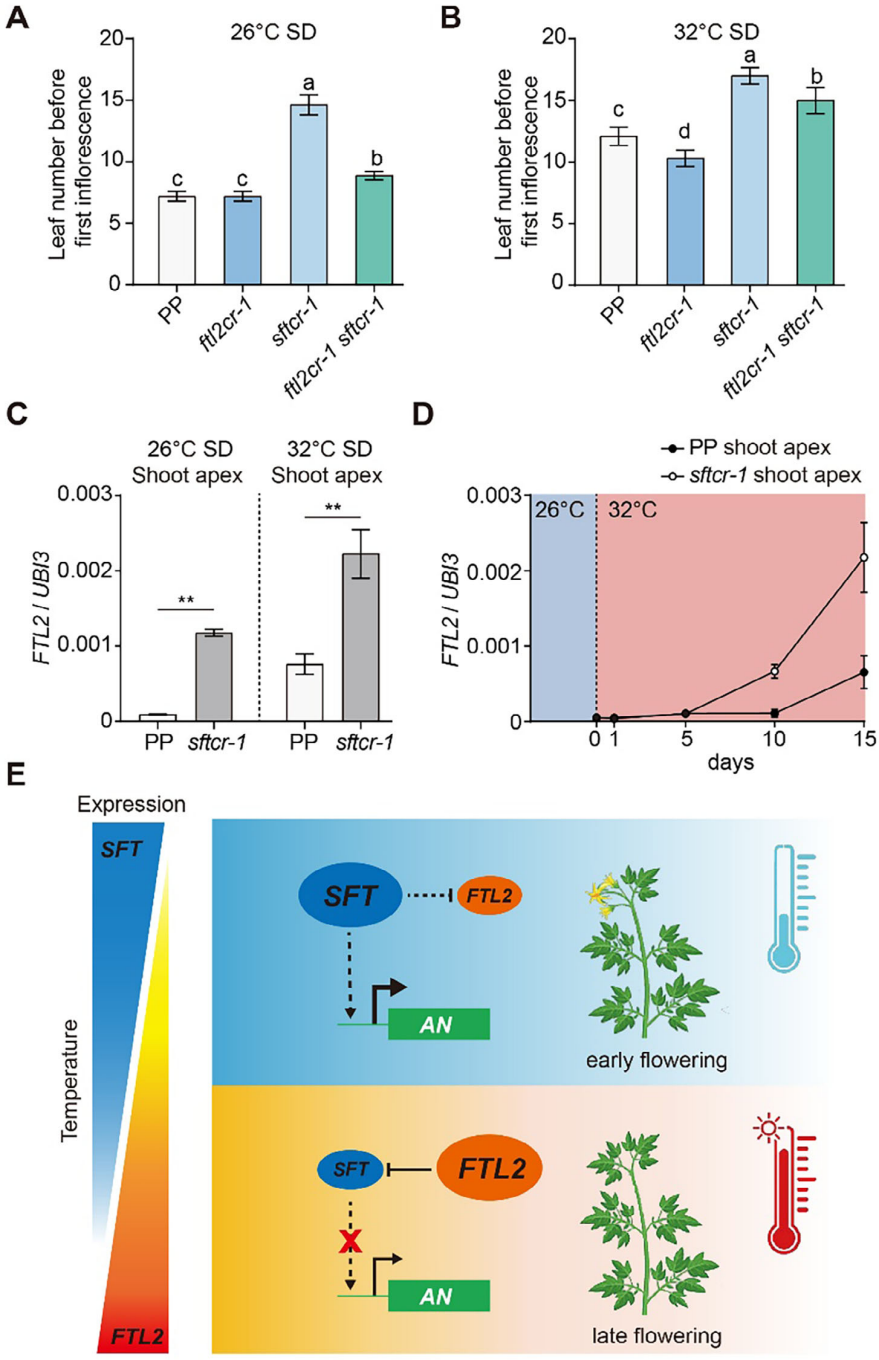
SFT represses *FTL2* expression in the shoot apex under SD conditions. A,B) Leaf number before the first inflorescence in PP, *ftl2cr‐1*, *sftcr‐1*, and *ftl2cr‐1 sftcr‐1* mutants grown under 26 °C‐SD (A) and 32 °C‐SD (B) conditions. Data are presented as means ± s.d. (*n*  =  10–16). Different lowercase letters denote statistically significant differences (*p* <0.05), as determined by one‐way ANOVA followed by Tukey's multiple comparisons test. C) Relative *FTL2* expression level in shoot apical meristems of PP and *sftcr‐1* plants at 12 DAG under 26 °C‐SD conditions and at 25 DAG under 32 °C‐SD conditions. Expression levels were normalized to *UBI3*. Data are presented as mean ± s.d. (*n*  =  3). Data were compared by a two‐tailed Student's *t*‐test, ^**^ *p* <0.01. D) Relative temporal expression of *FTL2* in shoot apical meristems of PP and *sftcr‐1* mutant after temperature shifts. Seedlings were grown for 5 DAG at 26 °C, then transferred to 32 °C for 0, 1, 5, 10, or 15 days. Samples were collected at the specified time points. Expression levels were normalized to *UBI3*. Data are presented as mean ± s.d. (*n * =  3). E) The proposed working model illustrates the roles of FTL2 and SFT in regulating flowering time at both normal and high temperatures under SD conditions.

Since our results demonstrate that FTL2 and SFT are important regulators of tomato flowering under high‐temperature conditions, we subsequently generated *ftl2cr‐1 sftcr‐1* double mutant via hybridization and observed their flowering phenotypes under 26 °C‐ and 32 °C‐SD conditions. Although the *ftl2cr‐1* single mutant displayed no apparent flowering phenotype, it significantly rescued the delayed flowering phenotype of *sftcr‐1* at 26 °C (**Figure** **6**A). However, at 32 °C, the early flowering phenotype of *ftl2cr‐1* was markedly suppressed by the *sftcr‐1* mutant (Figure 6B). This temperature‐dependent phenotypic divergence in the double mutant suggests that FTL2 accumulation at the shoot apex is the primary driver of delayed flowering in *sftcr‐1* under normal temperatures. However, FTL2 is transcriptionally activated under elevated temperatures and exerts an inverse regulatory effect, actively repressing SFT function.

To elucidate the molecular basis of these genetic interactions, we investigated whether SFT modulates *FTL2* expression in shoot apices. Quantitative analysis revealed significant upregulation of *FTL2* expression in *sftcr‐1* mutants under both 26 and 32 °C‐SD conditions compared to wild‐type PP (Figure 6C), demonstrating that SFT normally suppresses *FTL2* transcription. Notably, when plants acclimated to 26 °C were transferred to 32 °C, the thermal induction of *FTL2* expression in shoot apices was significantly amplified in *sftcr‐1* mutants compared to PP controls (Figure 6D). These results establish *SFT* as a key repressor of temperature‐induced *FTL2* accumulation. Taken together, these findings demonstrate a reciprocal regulatory relationship between *FTL2* and *SFT*, with SFT suppressing *FTL2* expression under normal conditions and FTL2, in turn, repressing *SFT* expression under high‐temperature conditions (Figure 6E). This dynamic interaction is crucial for maintaining flowering time homeostasis, ensuring precise developmental timing in response to ambient temperature fluctuations.

## 3 Discussion

Plants optimize reproductive success by integrating seasonal cues such as temperature and photoperiod to precisely regulate flowering time. In many species, the transition from vegetative to reproductive growth is predominantly controlled by the expression of the *FT* gene, which encodes a long‐distance mobile florigen transport from leaves to the shoot apex to induce flowering. In this study, we identified a unique regulatory module in tomato that involves two FT homologs, FTL2 and SFT. Unlike classical florigens expressed in leaves, *FTL2* and *SFT* are expressed in the meristem, where their expressions are directly influenced by ambient temperature. Furthermore, the reciprocal regulation between *FTL2* and *SFT* enables plants to sense and adapt to temperature fluctuations, ensuring that flowering occurs under optimal conditions. This study highlights how temperature‐responsive interactions within the FTL2‐SFT module contribute to the precise timing of the reproductive transition.

Plants have evolved distinct strategies to cope with high temperatures, driven by differences in their life cycles and reproductive priorities. Annual plants, like *Arabidopsis*, complete their life cycle within a single growth season, making successful reproduction their primary goal. In response to high temperatures, annual plants often accelerate their development, including flowering and seed production, to escape stress and ensure reproduction, even at the cost of reduced biomass and yield. In contrast, perennials, such as tomato, prioritize long‐term survival and fitness. These plants delay flowering under high temperatures to optimize the timing of reproduction (Figure 1A,B), allowing for the strategic allocation of resources between immediate growth, reproduction, and storage to support future growth cycles. By balancing short‐term and long‐term objectives, tomatoes can better withstand environmental stress and ensure reproductive success over multiple years. These contrasting strategies highlight the remarkable adaptability of plants to diverse environmental challenges.

The duplication and functional diversification of *FT* genes are crucial for balancing reproductive and vegetative growth in diverse plant species. These FT paralogs have evolved to perform antagonistic functions and respond to distinct environmental signals, enabling plants to fine‐tune their developmental processes.^[^ ^23^, ^51^, ^52^, ^53^ ^]^ The divergence in expression pattern and functional differentiation among *FT*‐like genes provides an adaptive strategy for sensing seasonal cues, facilitating the evolution of complex traits that optimize plant growth and reproduction in changing environments. In tomato, the FT paralogs FTL1 and SP5G play roles in sensing LD and SD photoperiod signals, with allelic variations of these genes controlling day‐neutral flowering.^[^ ^25^, ^26^, ^27^ ^]^ In this study, we identified another FT paralog FTL2, which specifically responds to ambient temperature signals in the shoot apex and repressed *SFT* expression under high temperatures. This regulation establishes a temperature‐dependent balance between FTL2 and SFT. Notably, our findings suggest that FTL2 likely regulates additional flowering‐related genes beyond *SFT*, as *sftcr‐1* partially rescued the early flowering phenotype of *ftl2cr‐1* but not completely (Figure 6B). At normal temperatures, SFT maintains *FTL2* expression at low levels to ensure timely flowering. However, under high temperatures, the interaction between *FTL2* and *SFT* delays flowering, allowing plants to flower in more favorable conditions (Figure 6E). This dynamic regulatory interplay highlights the sophisticated strategies for plants to adapt flowering time to environmental cues.

Our finding highlights the critical role of meristem‐specific regulation of florigen‐like components in modulating flowering time and plant development in response to environmental cues. FT in *Arabidopsis* and its homolog SFT in tomato serve as central regulators of ambient sensing, as evidenced by the temperature‐insensitive phenotypes observed in both overexpression lines and loss‐of‐function mutants (Figure 2A; Figure 5G).^[^ ^15^, ^16^, ^19^ ^]^ This shared characteristic illustrates the conserved role of *FT*‐like genes as central players in thermosensing across plant species. In tomato, however, floral induction relies on both *SFT* synthesized in the leaves and its self‐activation within the shoot apex (Figure 5). Without the activation of *SFT* in the shoot apex, leaf‐derived signals including SFT are insufficient to promote flowering, as demonstrated by the inability to complement the *sft* mutant phenotype through grafting with wild‐type plants (Figure 5A–C).

Interestingly, this self‐activation mechanism in tomato parallels the regulatory mode of tuberization in potatoes. The tuberigen signal StSP6A in potatoes, a homolog of FT, propagates in the stolon to promote tuber formation.^[^ ^54^ ^]^ Grafting experiments with *StSP6A*‐RNAi stocks revealed a delayed onset of tuberization, indicating that a high concentration of StSP6A in the stolon is essential for initiating the developmental transition.^[^ ^32^ ^]^ This critical requirement for a high level of *SFT* or *StSP6A* in the meristem or stolon likely reflects the need to overcome strong local repressive signals. This dynamic balance between promoting and repressive signals in the shoot apex or stolons enables plants to begin repeated transitions between vegetative and reproductive growth states. These mechanisms represent an evolutionary adaptation of complex perennial traits, allowing plants to repeatedly transition between developmental phases in response to environmental and physiological cues.

High‐temperature stress is a significant challenge to global crop productivity, necessitating the development of resilient crops that balance high yield under optimal conditions with stable performance under stress.^[^ ^2^, ^55^ ^]^ Our findings establish FTL2 as a pivotal regulator of ambient temperature sensing, modulating flowering time by repressing *SFT* expression under high temperatures. This regulatory mechanism highlights *FTL2* as a valuable biomarker for the strategic design of “smart crops” capable of balancing floral induction with environmental adaptation. By integrating *FTL2*‐*SFT* dynamics, this approach offers a promising avenue for enhancing crop resilience and stabilizing food security in the face of rising global temperatures.

## 4 Experimental Section

### Flowering Time Measurement and Growth Conditions

To assess flowering time, seeds were germinated on moistened filter paper in Petri dishes at 28 °C. Seedlings at comparable germination stages were transplanted into a soil mixture (peat:vermiculite:perlite = 2:1:1) and grown in a growth chamber under two temperature regimes: normal (26 °C light/22 °C dark) and high (32 °C light/28 °C dark), combined with either short‐day (8 h light/16 h dark) or long‐day (16 h light/8 h dark) photoperiods. Flowering time was quantified by counting the number of leaves preceding the first inflorescence on the primary shoot of a minimum of 10 individual plants. Data are presented as means ± standard deviation (s.d.). For the shift experiments, plants were first cultivated at 26 °C for 5 days and then transferred to 32 °C for incremental periods of 1, 5, 10, and 15 days.

### Constructs and Plant Transformation

For the CRISPR/Cas9 constructs, two sgRNA binding sites were selected for each target gene using the CRISPR‐P v2.0 tool (http://cbi.hzau.edu.cn/CRISPR2/). Primers incorporating sgRNAs and BsaI recognition sites were used to amplify the U6‐26t_SlU6p_sgRNA fragments, with the pCBC_DT1T2_SlU6p vector serving as the template. The amplified fragments were purified and subsequently cloned into the pTX041 vector at the BsaI recognition sites.^[^ ^27^, ^56^ ^]^ The plasmids were verified by Sanger sequencing and transformed into the *Agrobacterium tumefaciens* strain *AGL1*. The CRISPR/Cas9 vector was transformed into wild‐type PP by the leaf disc transformation method. Genomic DNA extracted from T0‐positive plants served as templates to amplify fragments encompassing the target sites, which were then subjected to Sanger sequencing to identify homozygous genome‐edited lines.

The *SFT::gSFT‐HA* complementation vector was constructed using the native *SFT* promoter and the full‐length genomic DNA of *SFT*. The promoter and gDNA fragment, amplified using wild‐type PP genomic DNA as the template, were cloned into the *pCAMBIA2300‐HA* vector utilizing the In‐Fusion cloning system kit (Clontech, 639648). The *SFT::gSFT‐HA* complementation vector was introduced into the *sftcr‐1* mutant via Agrobacterium (*AGL1*)‐mediated transformation. The transgenic lines were confirmed by PCR analysis.

To generate the misexpressor lines, the genomic DNA sequences of either *SFT* or *FTL2* were cloned downstream of the *TCS* promoter or the *SUC2* promoter and inserted into the *pCAMBIA2300‐HA* vector utilizing the In‐Fusion cloning system kit (Clontech, 639648). The *TCS::gSFT‐HA* plasmid was introduced into the *sftcr‐1* mutant via Agrobacterium (*AGL1*)‐mediated transformation, whereas the *TCS::gFTL2‐HA* and *SUC2::gFTL2‐NLS* plasmids were introduced into the *ftl2cr‐1* mutant employing an identical transformation protocol. The transgenic lines were subsequently validated through PCR analysis to confirm the successful integration of the constructs. All primers used are listed in Table S3 (Supporting Information).

### Tissue Collection, RNA Extraction, and Gene Expression Analysis

Shoot apical meristems and leaves were collected on various days after germination (DAG) from plants grown at 26 and 32 °C under SDs and LDs. Total RNA was isolated using the TRNzol Universal reagent (Tiangen, DP424) following the manufacturer's instructions.

Meristems cultivated at 26 and 32 °C under SD conditions were collected within 2 h and promptly fixed in 100% acetone, followed by vacuum infiltration (0.06 MPa, 40 min) as previously described.^[^ ^33^ ^]^ Meristem tissues (VM, TM, SIM, FM, and SYM) were microdissected using fine tweezers under a stereomicroscope, air‐dried for 3 min at room temperature to eliminate residual acetone, and subsequently flash‐frozen in liquid nitrogen. Total RNA was purified from each sample using the PicoPure RNA Isolation Kit (Thermo Fisher Scientific, 12204‐01).

Reverse transcription was performed using the TransScript II One‐Step gDNA Removal and cDNA Synthesis SuperMix (TransGen, AH311‐02), with 500 ng of total RNA as the template. Quantitative PCR (qPCR) was performed using the Taq Pro Universal SYBR qPCR Master Mix (Vazyme, Q712‐02) on a Bio‐Rad CFX‐96 Real‐Time PCR Detection System. The thermal cycling protocol included an initial denaturation step at 95 °C for 3 min, followed by 40 cycles of denaturation at 95 °C for 20 s, annealing at 60 °C for 30 s, and extension at 72 °C for 20 s. The *UBI3* gene (*Solyc01g056840*) was utilized as an internal control for qRT‐PCR normalization. The primer sequences employed for qRT‐PCR are provided in Table S3 (Supporting Information).

### In Situ Hybridization Assay

In situ hybridization was performed as described in Wang et al. with modifications.^[^ ^57^ ^]^ The cDNA fragments of *SFT* and *FTL2* were amplified using primers P22 and P23, respectively (Table S3, Supporting Information), and subsequently cloned into the pEAZY‐T3 vector (TransGen, CB301‐01), which contains T7 promoter sequences. In vitro transcription was carried out using T7 RNA polymerase to generate both antisense and sense RNA probes for in situ hybridization. Shoot apical meristems were harvested at 6 and 12 DAG under 26°C‐ and 32 °C‐SD conditions, and fixed for 12–16 h at 4 °C in freshly prepared 4% (w/v) paraformaldehyde in phosphate‐buffered saline (PBS, pH 7.2). Fixed tissues were dehydrated through a graded ethanol‐HistoChoice (Sigma–Aldrich, H2779) series and subsequently embedded in Paraplast wax (Leica, 39 601 006). Dewaxed tissue sections (8 µm in thickness) were hybridized with hydrolyzed RNA probes for 12 h at 50 °C. Color development was carried out, and the sections were examined under a bright‐field microscope (Olympus, Model BX43).

### Grafting Experiments

Scions for grafting were confirmed to be at the VM stage at 15 DAG. A wedge‐shaped/slit grafting technique was employed, with the graft union secured using a grafting clip. Grafted plants were maintained under shaded, high‐humidity conditions (80% RH) for 3‐5 days. Successfully grafted plants were then transferred to 26 °C short‐day (SD) conditions for subsequent growth. When SAMs in the control group scions during floral transition, SAMs from all grafted plant scions were collected. These samples were subjected to qRT‐PCR analysis to detect transmission of *SFT* or *SFT‐HA* mRNA. Flowering time was assessed by counting the number of leaves preceding the first inflorescence on the primary shoot. All primers used are listed in Table S3 (Supporting Information).

### Western Blot

*TCS::gFTL2‐HA ftl2cr‐1* plants grown at 26 °C for 5 days, after which they were either transferred to 32 °C or maintained at 26 °C for an additional 5 and 10 days. The shoot apices of *TCS::gFTL2‐HA ftl2cr‐1* plants were harvested at various time points. Total proteins were extracted using IP‐extraction buffer (50 mm Tris‐HCl, pH 7.5; 150 mm NaCl; 0.19% (v/v) CA630; 20% (v/v) glycerol; 5 mM DTT; and 1 tablet/50 mL of protease inhibitor cocktail) and subsequently incubated overnight at 4 °C with HA‐binding beads (5 µg anti‐HA antibody (Sigma, H6908) conjugated to 20 µL Dynabeads Protein G (Novex, 10001D). The proteins were separated via electrophoresis on a 15% SDS–PAGE gel and subsequently transferred to a PVDF membrane (Immobilon‐P, IPVH00010). Immunoblotting was conducted using an anti‐HA antibody (Sigma, H6958) at a dilution of 1:2000. The protein bands were detected using a Tanon‐5200 Chemiluminescent Imaging System (Tanon Science and Technology).

### Cell Fractionation Assay

Shoot apex of *TCS::gFTL2‐HA ftl2cr‐1* plants grown at 26 and 32 °C under SDs were ground in liquid nitrogen, and then homogenized in ice‐cold nuclei isolation buffer (1 M sucrose; 10 mm HEPES, pH 7.6; 5 mm MgCl_2_; 5 mm KCl; 5 mm EDTA, pH 8.0; and 1 tablet/50 mL of protease inhibitor cocktail (Roche, 04693132001). The homogenate was mixed thoroughly and centrifuged at 1,260 g for 10 min at 4 °C. The supernatant was discarded, and the pellet was resuspended in nuclei isolation buffer containing 0.6% Triton‐X‐100 and centrifuged at 12000 g for 10 min at 4 °C. The supernatant (cytoplasm) was collected, and the pellet (nucleus) was resuspended in nuclei isolation buffer containing 0.6% Triton‐X‐100. The supernatant nuclei pellet was placed in nuclei separation solution (1 M sucrose; 10 mm HEPES, pH 7.6; 5 mm MgCl_2_; 5 mm KCl; 5 mm EDTA, pH 8.0; 15% Percoll, and 1 tablet/50 mL of protease inhibitor cocktail), and centrifuged at 3000 *g* for 10 min at 4 °C. The supernatant was removed and the precipitate was the nucleus. All protein samples were subjected to immunoprecipitation (IP) with anti‐HA (Sigma, H6908) followed by Western blotting. Anti‐HSP70 (Agrisera, AS08 371) and anti‐H3 (Abcam, ab1791) antibodies were used as cytoplasmic and nuclear markers, respectively.

### RNA‐seq Analysis

PP and *ftl2cr‐1* plants were initially cultivated at 32 °C for 12 days, after which they were either transferred to 26 °C or maintained at 32 °C for an additional 8 days. Total RNA was isolated from the shoot apices of PP and *ftl2cr‐1* plants at 20 DAG. Three independent biological replicates were conducted. A total of nine RNA‐Seq libraries were constructed and sequenced using the Illumina HiSeq2000 platform at Berry Genomics (http://www.berrygenomics.com/). The filtered clean reads were aligned to the tomato genome (ITAG4.0) using STAR v2.5.3, and their features were quantified using featureCounts v1.5.3, as previously described.^[^ ^57^ ^]^ The statistical package DEGseq, employing the MA‐plot‐based method in R version 3.0.3, was utilized to calculate P values, which were subsequently adjusted using the Benjamini–Hochberg procedure. The fold changes between PP_26 °C_SD and PP_32 °C_SD libraries, as well as between PP_32 °C_SD and *ftl2cr‐1*_32 °C_SD libraries, were calculated based on FPKM (fragments per kilobase of transcript per million mapped reads). The thresholds for identifying differentially expressed genes (DEGs) were set as follows: a fold change >1.5 or <0.667, and a Benjamini–Hochberg adjusted P value (padj) <0.01. Gene Ontology (GO) analyses of the overlapping DEGs were conducted using their *Arabidopsis* homologs via DAVID (The Database for Annotation, Visualization, and Integrated Discovery; https://david.ncifcrf.gov/).

### ChIP and ChIP‐qPCR

ChIP was performed using 0.5 g of shoot apical meristems from *TCS::gFTL2‐HA ftl2cr‐1* plants grown at 26 and 32 °C under SD conditions, as described previously.^[^ ^58^ ^]^ SAMs were thoroughly ground in liquid nitrogen and cross‐linked in 1% (v/v) formaldehyde (Sigma–Aldrich) for 10 min at 4 °C. Chromatin was sheared using a Diagenode Bioruptor Plus instrument to obtain ≈300‐bp DNA fragments. Anti‐HA antibody (Sigma, H6908) was used for immunoprecipitation. DNA isolated by ChIP was used for qPCR analysis. ChIP‐qPCR was performed using Taq Pro Universal SYBR qPCR Master Mix (Vazyme, Q712‐02) on a Bio‐Rad CFX‐96 Real‐Time PCR instrument with the following program: 3 min at 95 °C, followed by 50 cycles of 20 s at 95 °C, 30 s at 60 °C, and 20 s at 72 °C. The intergenic region surrounding *ACTIN* (*Solyc03g078400*) was used as an internal control. Primers for qPCR are provided in Table S3 (Supporting Information).

### Phylogenetic Analysis

Protein sequences of *Arabidopsis*, tomato, and rice CETS family members were retrieved via BLASTP from the *Arabidopsis* Information Resource (TAIR) (https://www.arabidopsis.org/), the Sol Genomics Network (https://solgenomics.net/), and Rice Database Oryzabase (https://shigen.nig.ac.jp/rice/oryzabase/). Multiple sequence alignments of the full‐length proteins were performed using the ClustalW algorithm implemented in MEGA10. The phylogenetic tree was constructed in MEGA10 using the neighbor‐joining method with Poisson correction. Bootstrap analysis was performed with 1000 replicates. Positions with gaps and missing data were excluded from the analysis.

### Subcellular Localization

The *35S::FTL2‐eGFP* vectors were constructed by amplifying the CDSs of *FTL2* from PP cDNA and fusing them to the N‐terminus of eGFP. The plasmid was transformed into *Agrobacterium tumefaciens* strain EHA105, followed by co‐infiltration into the leaves of 4‐week‐old *Nicotiana benthamiana* along with an mCherry‐labeled nuclear marker, NLS‐mCherry. The injected tobacco was cultured for two days at 26 and 32 °C under SD conditions. Localization of the fluorescent fusion proteins was analyzed using confocal scanning laser microscopy (Leica DM6 CS). All primers used are listed in Table S3 (Supporting Information).

### Yeast Two‐Hybrid

The coding sequences for bait proteins (14‐3‐3 and SPGB proteins) were cloned into the pGBKT7 vector, and the coding sequences for prey proteins (FTL2 and FTL2^mu^ (F63L, Q64R, S98T) proteins) were cloned into the pGADT7 vector. A pair of bait and prey plasmids were co‐transformed into the Y2HGold yeast strain following the Clontech Yeast Protocol Handbook instructions. The resultant strains were subsequently grown on plates lacking leucine and tryptophan for 3 days at 30 °C. The interaction was tested via growth assays on a medium lacking leucine, tryptophan, and histidine, but containing 5 mm 3‐AT, over 3 days. All primers used are listed in Table S3 (Supporting Information).

## Conflict of Interest

The authors declare no conflict of interest.

## Author Contributions

J.S. carried out most of the experiments; S.Z. analyzed the RNA‐seq data; S.F. and S.S. assisted in completing the in situ experiments; X.W. and R.L. contributed to phenotype observations; X.C. and L.L. designed the research and wrote the article.

## Supporting information



Supporting Information

Supplemental Tables

## Data Availability

The RNA sequencing datasets generated in this study have been deposited in the Sequence Read Archive (SRA) under the accession number PRJNA1222593. Other data supporting the findings are available in the manuscript file or from the corresponding author upon request.
