# Supplementary material for: Florigen and Florigen‐Like Genes Regulate Temperature‐Responsive Flowering in Tomato
Source: Adv Sci (Weinh). 2025 Jul 24;12(39):e06711. doi: 10.1002/advs.202506711 (PMC12533152; doi:10.1002/advs.202506711)
Supplement: Supplementary file 1 — Supporting Information [file ADVS-12-e06711-s001.pdf]

ADVANCED SCIENCE
Open Access

# Supporting Information

for *Adv. Sci.*, DOI 10.1002/advs.202506711

Florigen and Florigen-Like Genes Regulate Temperature-Responsive Flowering in Tomato

*Jia Song, Shiqi Zhao, Siyu Fang, Xiaotian Wang, Shuai Sun, Baohua Li, Ren Li, Lu Liu* and Xia Cui**

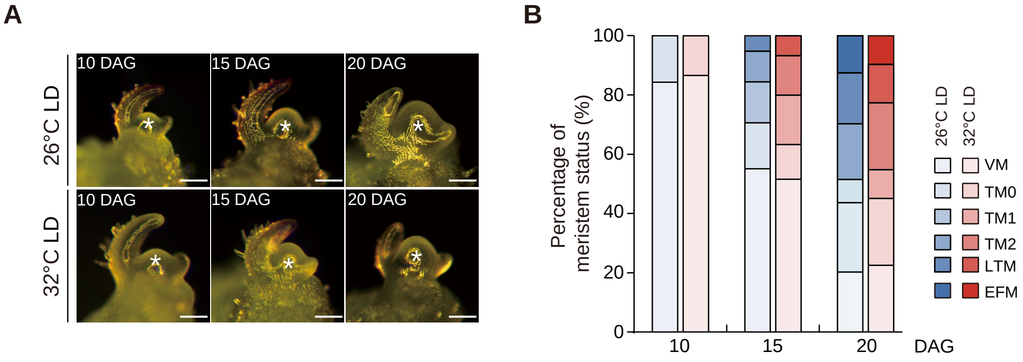


**Figure S1. High temperature does not regulate flowering time in tomato under LD conditions.**
**A)** Representative images of shoot apical meristem during the floral transition under 26°C-LD and 32°C-LD conditions. DAG: Days after germination. Scale bar, 50 µm.
**B)** Percentage of meristem status at 10, 15, and 20 DAG under 26°C-LD and 32°C-LD conditions ($n$ = 55–65). Meristem maturation is characterized by six sequential stages of primary shoot meristem development: VM, TM0, TM1, TM2, LTM, and EFM.

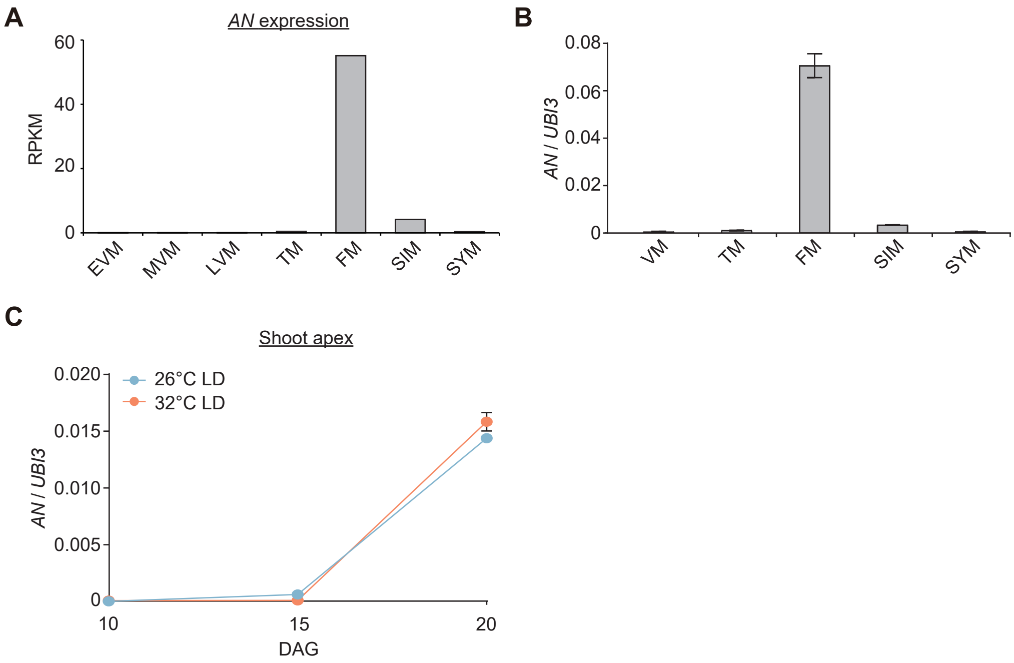


**Figure S2. *AN* is specifically expressed in the floral meristem and concurrently induced under 26°C-LD and 32°C-LD conditions.**

**A)** RPKM values of *AN* in five stages of primary shoot meristem maturation. EVM, early vegetative meristem; MVM, middle vegetative meristem; LVM, late vegetative meristem; RPKM, reads per kilobase per million mapped reads. Data were obtained from Park et al. (2012).

**B)** qRT-PCR analysis of *AN* expression in different meristematic tissues of PP.

**C)** Temporal expression profiles of *AN* in the shoot apical meristems of developing PP seedlings under 26°C-LD and 32°C-LD conditions. Expression levels in **B** and **C** were normalized to *UBI3*, and data are presented as mean ± s.d. ($n = 3$).

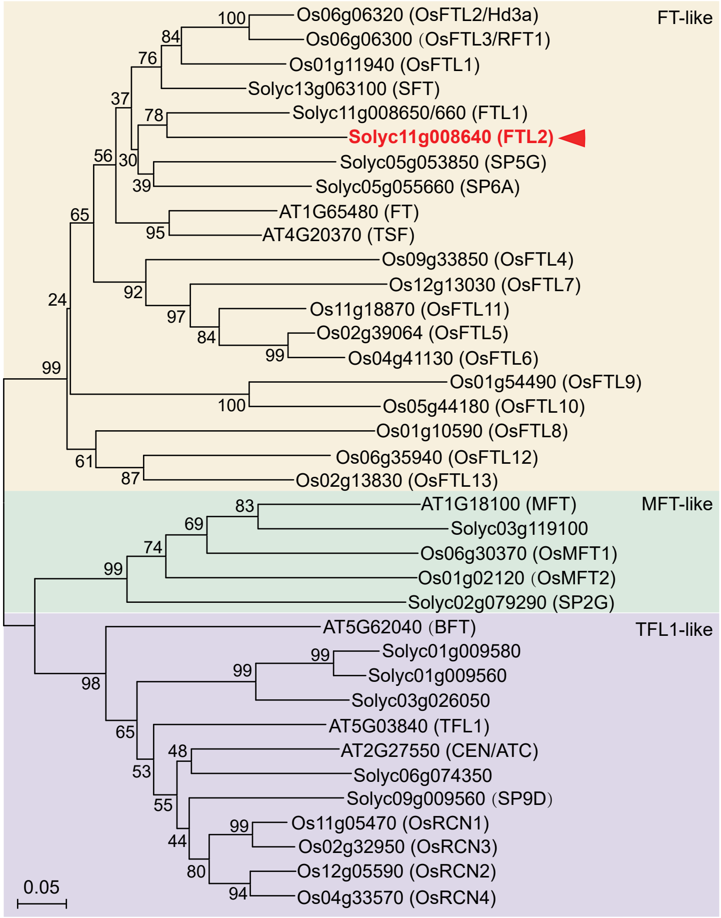


**Figure S3. Phylogenetic analysis of the CENTRORADIALIS/TERMINAL FLOWER 1/SELF-PRUNING (CETS) family proteins in** ***Arabidopsis*****, tomato and rice.**
Phylogenetic tree of CETS family proteins in *Arabidopsis*, tomato and rice showing three clades: FLOWERING LOCUS T (FT), MOTHER OF FT (MFT), and TERMINAL FLOWER 1 (TFL1).

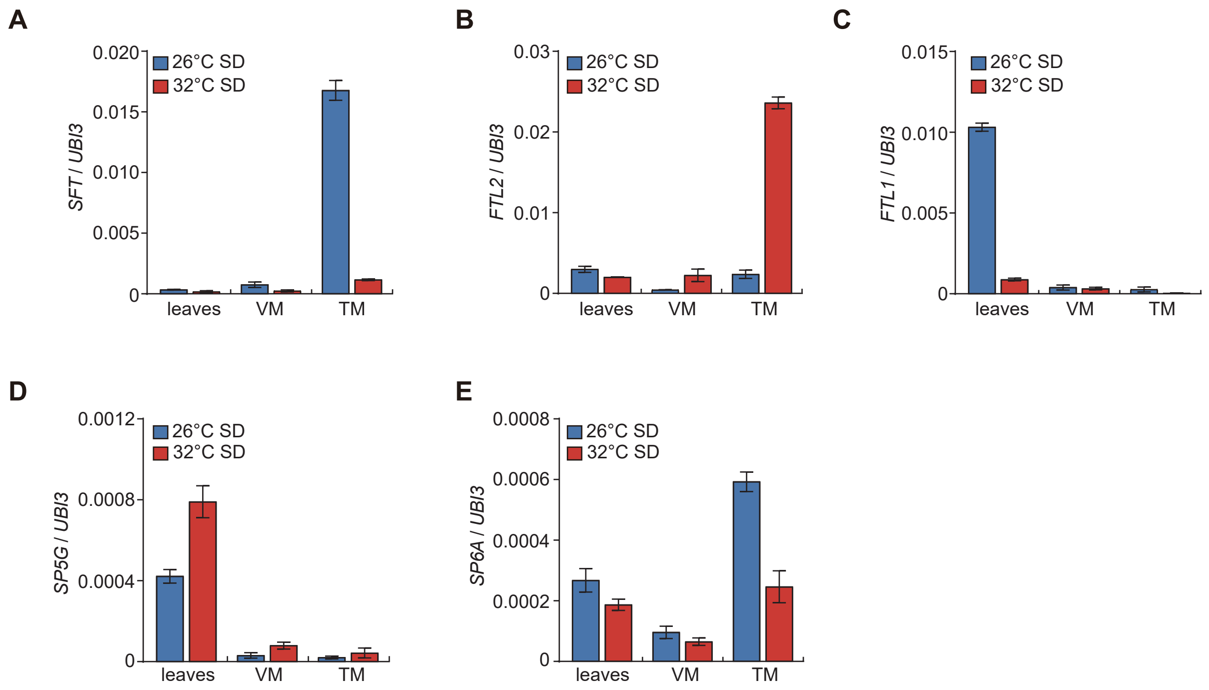


**Figure S4. Relative expression of five *FT*-like genes in leaves, VM, and TM.**
Relative expression of *SFT* **(A)**, *FTL2* **(B)**, *FTL1* **(C)**, *SP5G* **(D)**, and *SP6A* **(E)** in leaves, VM, and TM under 26°C-SD and 32°C-SD conditions. Expression levels were normalized to *UBI3*. Data are presented as mean ± s.d. ($n = 3$).

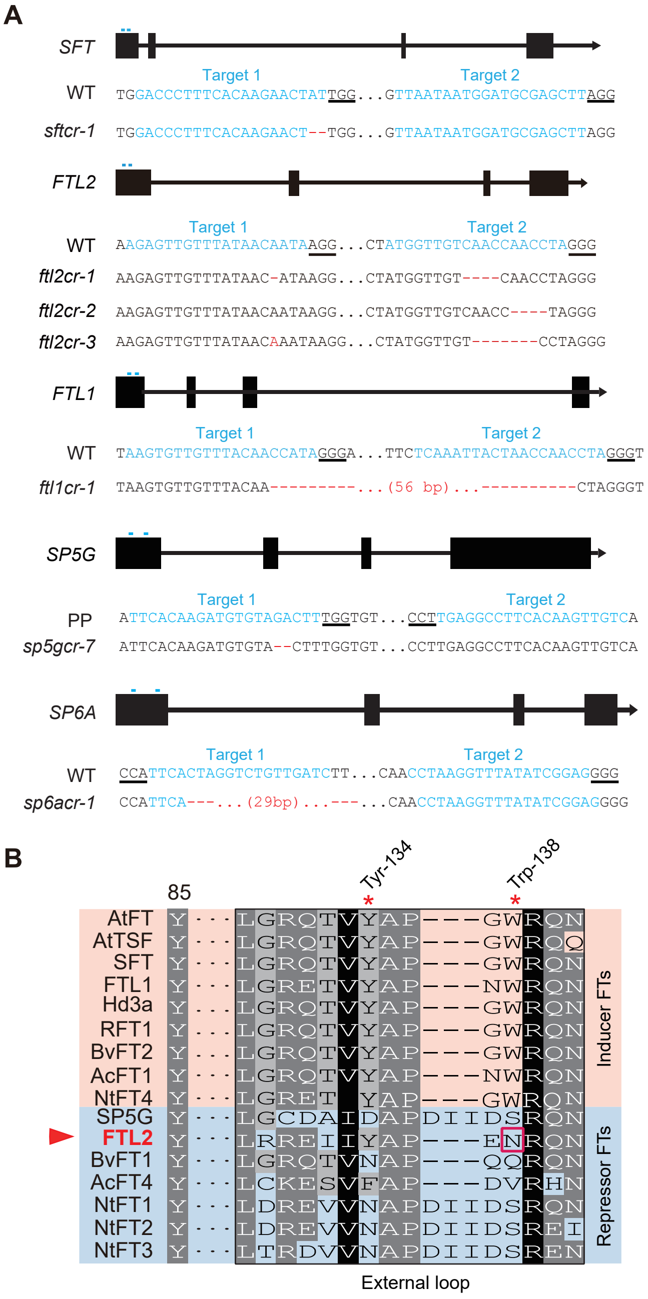


**Figure S5. FTL2 functions as a floral repressor.**
**A)** The *sft*, *ftl2*, *ftl1*, *sp5g*, and *sp6a* knockout mutants were generated through CRISPR/Cas9-mediated genome editing in the wild-type PP background. Target sequences are highlighted in blue. Deletion or insertion sequences are indicated by red dashed lines or red text. The protospacer-adjacent motif (PAM) is underlined in black.
**B)** Partial alignment of FT family proteins from *Arabidopsis*, tomato, rice, sugar beet, onion, and tobacco. The external loop domain that confers flower promoting and repressing activity is highlighted in a black box. Previous studies defined residues within the external loop domain that are decisive for florigenic activity, and the amino acid changes W138N is highlighted in a red box.

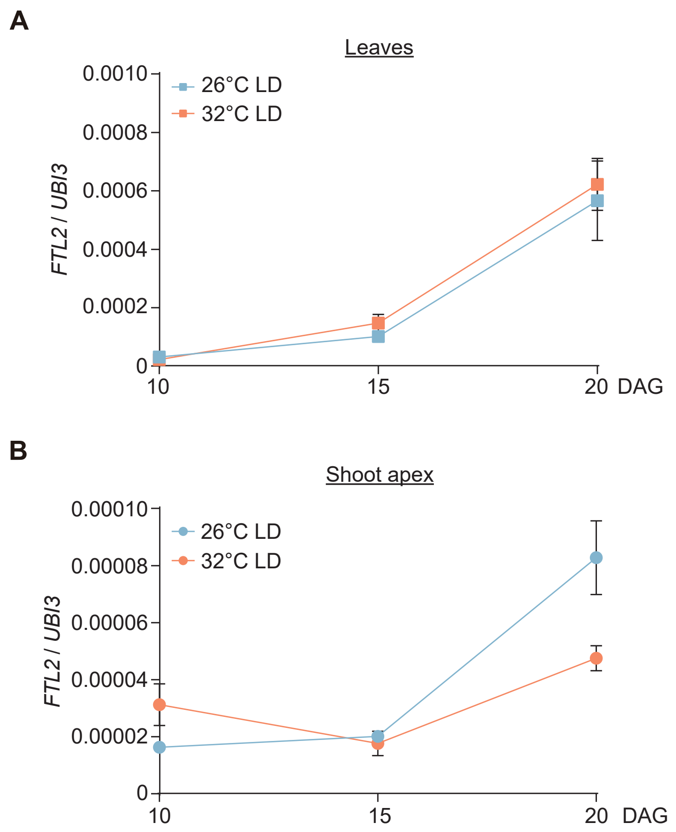


**Figure S6. High temperature does not induce the expression of *FTL2* under LD conditions.**
Relative temporal expression of *FTL2* in leaves **(A)** and shoot apical meristems **(B)** of developing PP seedlings under 26°C-LD and 32°C-LD conditions. Expression levels were normalized to *UBI3*. Data are presented as mean ± s.d. ($n = 3$).

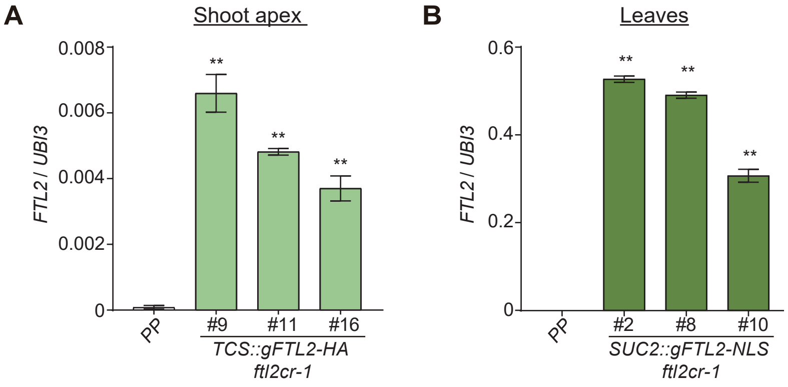


**Figure S7. *FTL2* is overexpressed in both *TCS::gFTL2-HA ftl2cr-1* and *SUC2::gFTL2-HA ftl2cr-1* transgenic lines.**
**A)** Relative expression of *FTL2* in shoot apical meristems of *TCS::gFTL2-HA ftl2cr-1*.
**B)** Relative expression of *FTL2* in leaves of *SUC2::gFTL2-NLS ftl2cr-1*. Expression levels in **A** and **B** were normalized to *UBI3*. Data are presented as mean ± s.d. ($n = 3$). Data in **A** and **B** were compared by two-tailed Student's *t*-test, $^{**}P < 0.01$.

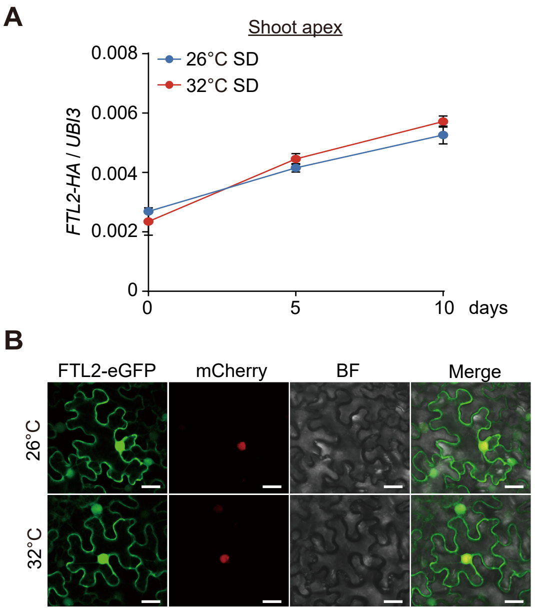


**Figure S8. High temperature does not affect FTL2-HA transcript and protein levels under SD conditions.**
**A)** *FTL2-HA* mRNA expression in shoot apical meristems of *TCS::gFTL2-HA ftl2cr-1* after temperature shifts. Seedlings were grown for 5 days at 26°C, then transferred to 32°C for 0, 5, and 15 days. Control seedlings were maintained at 26°C. Samples were collected and analyzed at the indicated time points. Expression levels were normalized to *UBI3*. Data are presented as mean ± s.d. ($n = 3$).
**B)** Subcellular localization of FTL2-eGFP proteins in *N. benthamiana* leaf epidermal cells under 26°C-SD and 32°C-SD conditions. An mCherry-labeled fusion protein (NLS-mCherry) was used as a nuclear marker. eGFP, green fluorescence; mCherry, red fluorescence; BF, bright field image; Merge, overlay of eGFP, mCherry and BF. Scale bars: 50 μm.

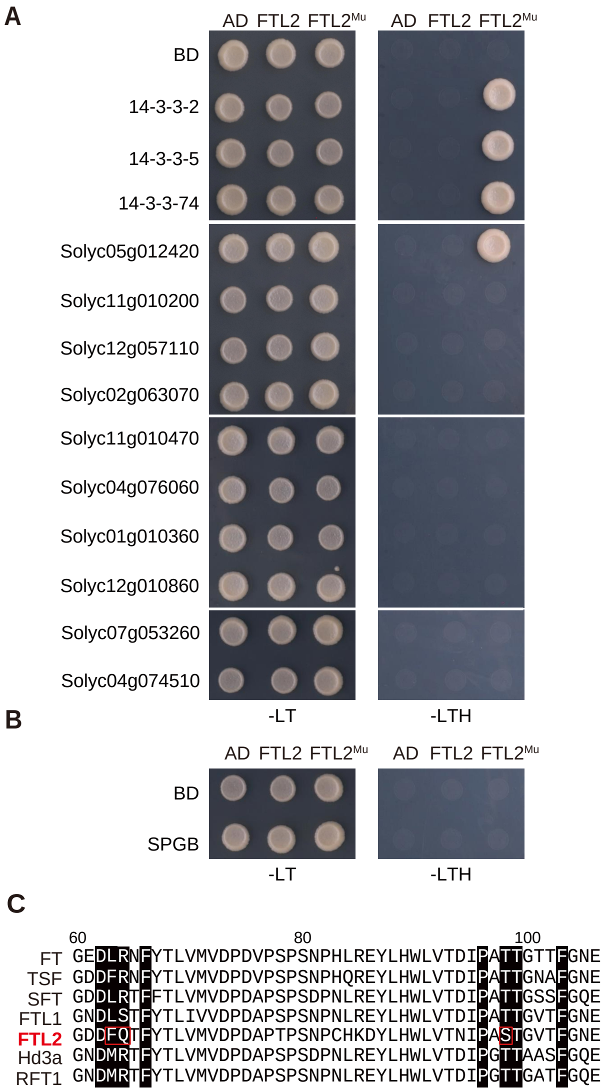


**Figure S9. FTL2 protein does not interact with 14-3-3 and SPGB.**

**A, B)** Yeast two-hybrid assays showing FTL2 protein does not interact with 14-3-3 and SPGB, whereas FTL2$^{Mu}$ (F63L, Q64R, S98T) protein interacts with 14-3-3. L, leucine; T, tryptophan; H, histidine.

**C)** Partial alignment of FT family proteins. Residues shown in white characters on a black background are located at the binding interface with 14-3-3 proteins, and are highly conserved in FT families, indicating the possibility that both FT family proteins bind 14-3-3 proteins. However, several of those amino acids are not conserved between FT family members and FTL2. The amino acid changes L63F, R64Q, and T98S are highlighted in red boxes.

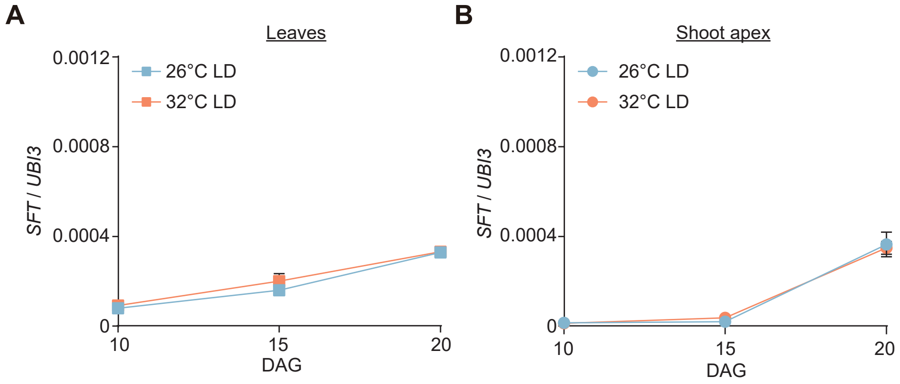


**Figure S10. High temperature does not suppress *SFT* expression under LD conditions.**
**A, B)** Relative temporal expression of *SFT* in leaves **(A)** and shoot apical meristems **(B)** of developing PP seedlings under 26°C-LD and 32°C-LD conditions. Expression levels were normalized to *UBI3*. Data are presented as mean ± s.d. ($n = 3$).

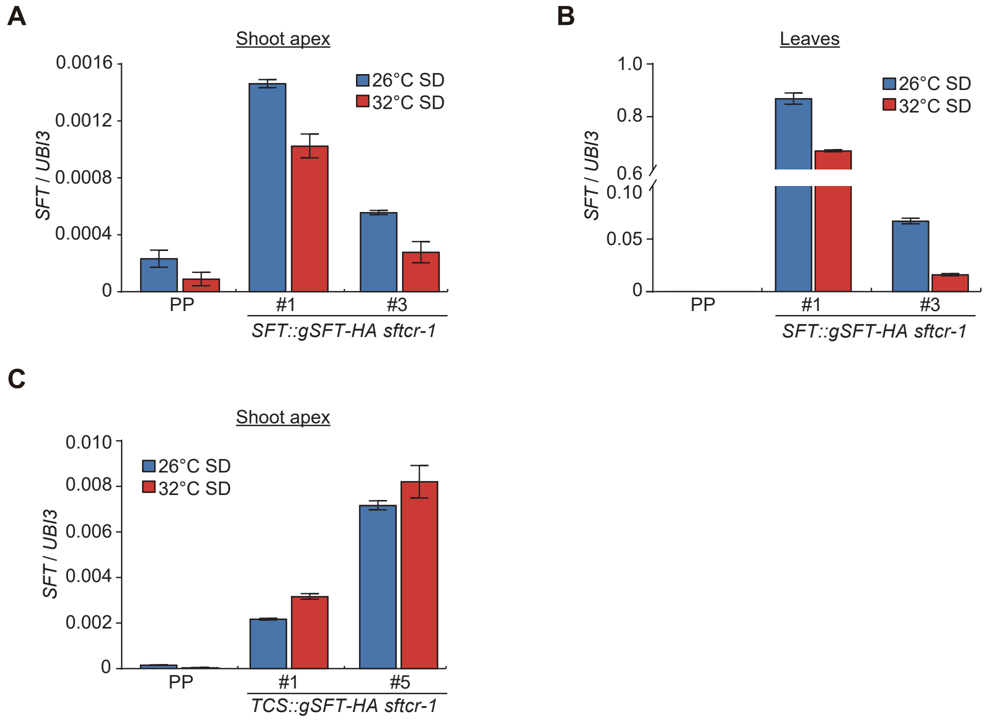


**Figure S11. *SFT* is overexpressed in both *SFT::gSFT-HA sftcr-1* and *TCS::gSFT-HA sftcr-1* transgenic lines.**
**A**, **B)** Relative expression of *SFT* in shoot apical meristems (**A**) and leaves (**B**) of *SFT::gSFT-HA sftcr-1* under 26°C-SD and 32°C-SD conditions.
**C)** Relative expression of *SFT* in shoot apical meristems of *TCS::gSFT-HA sftcr-1* under 26°C-SD and 32°C-SD conditions.
Expression levels in **A**, **B** and **C** were normalized to *UBI3*. Data are presented as mean ± s.d. ($n = 3$).
